# Detection of an arbitrary number of communities in a block spin Ising model

**DOI:** 10.1371/journal.pone.0339060

**Published:** 2026-03-17

**Authors:** Miguel Ballesteros, Ramsès H. Mena, Josè Luis Pèrez, Gabor Toth

**Affiliations:** 1 IIMAS-UNAM, Mexico City, Mexico; 2 CIMAT, Guanajuato, Mexico; Federal University of Pernambuco: Universidade Federal de Pernambuco, BRAZIL

## Abstract

We study the problem of community detection in a general version of the block spin Ising model featuring *M* groups, a model inspired by the Curie-Weiss model of ferromagnetism in statistical mechanics. We solve the general problem of identifying any number of groups with any possible coupling constants. Up to now, the problem was only solved for the specific situation with two groups of identical size and identical interactions, see [1, 2]. Our results can be applied to the most realistic situations, in which there are many groups of different sizes and different interactions. In addition, we give an explicit algorithm that permits the reconstruction of the structure of the model from a sample of observations based on the comparison of empirical correlations of the spin variables, thus unveiling easy applications of the model to real-world voting data and communities in biology.

## 1 Introduction and results

In the influential article [1], Berthet, Rigollet, and Srivastava introduced the block spin Ising model consisting of two groups previously defined in the statistical mechanics literature in [3] to the field of community detection, and the authors showed that for two groups of identical size and identical coupling constants within each group the exact recovery of the community structure was possible with high probability using a sample of observations assumed to be generated by the model. The number of observations required depends on the regime (or phase) of the model. Of the three regimes of the model, the high and low temperature regimes where analysed in [1]. Subsequently, Löwe and Schubert supplied the missing critical regime in [2], and demonstrated that exact recovery was possible there, too. Thus, the problem of community detection for more than two groups and/or different coupling constants remained open. We solve the problem for a wide variety of situations as far as the number of groups in the population, the sizes of the groups, and interactions between voters within each group and between different groups are concerned, and show that reconstructing the group structure is possible given a certain number of observations. The procedure we present is completely constructive and easy to implement. Thus, our results in the present article allow for the application of community detection algorithms to real-world data. Possible applications are the identification of social classes, cultural groups, or sympathisers of political parties, or other characteristics which may not be readily available in data. Using the community detection algorithm will potentially allow social scientists to better understand societal structures derived from common preferences or interdependencies. Biologists can employ the algorithms for the problem of distinguishing species based on the observation of characteristics of different specimens. An important point that should be noted is that the techniques presented in this article can in principle also be applied to any other voting model with several communities, for which the pair correlations between votes can be deduced.

The literature on the detection of communities in stochastic models is extensive. Among the models analysed, graphical models (i.e. Markov random fields) are most prominent. See [4] for a description of how Markov random fields encode dependencies between random variables. Among graphical models, the most studied are the stochastic blockmodel and the block spin Ising model. The stochastic blockmodel (introduced in [5]) has been analysed thoroughly. See [6] for an up to date exposition and [7–10] for further important results about this model. In the stochastic blockmodel, we observe a single realisation of the random graph and assume that interactions between individuals are independent. This is very different from the block spin Ising model which is the subject of the present article.

Beyond the mathematical interest inherent to these models and the problem of community detection, there are also important applications in the field of image recognition [11,12], biology and genetics [13–15], recommendation systems [16], and natural language processing [17], among others. These applications are especially important given the ubiquitous use of large amounts of data. Other applications include the use of these models applied to problems in social sciences, such as sociological behaviour [18], migration [3], economic decision making [19–21], and political science [22]. Community detection algorithms applied to these problems allow researchers to understand the socioeconomic and political structure of societies and dependencies between different groups and classes, as well as the structures underlying species diversity and interdependence.

Compared to the stochastic blockmodel, there has been considerably less work about the community detection problem in Block spin Ising models, also referred to as multi-species mean-field models [23] or multi-group Curie-Weiss models [24–26]. In these models, there is a heterogeneous population of N∈ℕ individuals subdivided into M∈ℕ groups and each individual casts a binary vote in an election. We can also interpret the binary choice as a biological characteristic of the individual which can be observed and measured. The votes or measurements are random variables which are allowed to depend on each other (with different degrees of dependencies within each group and across group boundaries). These dependencies are regulated by the coupling constants between votes. We will formally present the model in Sect 1.1. An observer has access to the votes of all individuals in a number of elections, referenda, or measurements. The population is assumed static, and the observer sees the patterns in the voting behaviour. These patterns manifest in the way certain subsets of voters frequently tend to vote alike or contrary to each other. The observer’s problem consists of reconstructing the group structure of the population from the observed votes or measurements alone. The method consists in calculating the empirical pair correlations between individual votes and grouping together those voters who present the highest correlations in the sample, i.e. those who tend to agree with the highest frequency are assumed to belong to the same community. See Sects 2.2 and 2.3 for a complete description of the algorithm involved in the reconstruction procedure.

Rather than taking a single realisation of a random graph as in the stochastic blockmodel, in the block spin Ising model we observe a sample of *n* realisations of the model, i.e. *n* voting configurations of the shape $\left(x_{1},\ldots ,x_{N}\right)\in {\left\{-1,1\right\}}^{N}$ which are realisations of the random vector $\left(X_{1},\ldots ,X_{N}\right)$ assumed to be independent and distributed according to the probability measure of the block spin Ising model defined in Definition 1.5. Each realisation of $\left(X_{1},\ldots ,X_{N}\right)$ contains the votes or measurements of the entire population in a single election, referendum, or observation. The problem we study consists of reconstructing the structure of the model in terms of assigning each of the random variables *X*_*i*_, $i\in {\mathbb{N}}_{N}$, (we will write ${\mathbb{N}}_{m}≔\left\{1,\ldots ,m\right\}$ for any m∈ℕ) that represents the vote of one individual to one of the *M* groups. Having access to a certain number of observations permits us to recover the structure with high probability for the possible structures of practical importance the model can assume.

### 1.1 The block spin Ising model

The block spin Ising model is defined for N∈ℕ{−1,1}-valued random variables *X*_*i*_ indexed by $i\in {\mathbb{N}}_{N}$, commonly referred to as spins. Since the application to voting and the observation of biological characteristics of a heterogeneous population is the main motivation of the study in this article, we will instead speak of votes and voting configurations. The voters are sorted into M∈ℕ groups. Each group $l\in {\mathbb{N}}_{M}$ is of size $N_{l}\in \mathbb{N}$, such that $\sum_{l=1}^{M}N_{l}=N$. The group identification function

$$\iota :{\mathbb{N}}_{N}\to {\mathbb{N}}_{M}$$ (1.1)

assigns each voter $i\in {\mathbb{N}}_{N}$ to their respective group $\iota (i)\in {\mathbb{N}}_{M}$. Hence, the definition of the group sizes above implies $\left|\iota^{-1}\left(\left\{l\right\}\right)\right|=N_{l}$, where for any countable set *A* the cardinality of *A* is denoted by |A|.

**Definition 1.1.** *We define the group size parameters for each group l*


$$\alpha_{l}:=\frac{N_{l}}{N}.$$

**Remark 1.2.** *We will assume these constants $\alpha_{l}$, $l\in {\mathbb{N}}_{M}$, exist and are positive. This implies in particular, that we can assume each group consists of at least two different individuals for large enough N, a fact we will use in the proofs of our results. While the population goes to infinity, the number of groups remains constant at M.*

For any voting configuration $\left(x_{1},\ldots ,x_{N}\right)\in {\left\{-1,1\right\}}^{N}$, the Hamiltonian $ℍ:{\left\{-1,1\right\}}^{N}\to \mathbb{R}$ is given by

$$ℍ\left(x_{1},\ldots ,x_{N}\right):=-\frac{1}{2}\sum_{l,m=1}^{M}\frac{J_{l,m}}{\sqrt{N_{l}N_{m}}}\sum_{i\in \iota^{-1}\left(\left\{l\right\}\right)}\sum_{j\in \iota^{-1}\left(\left\{m\right\}\right)}x_{i}x_{j}.$$ (1.2)

This Hamiltonian allows for different interactions between pairs of votes, such that if one belongs to group *l* and the other to group *m*, they interact by a coupling constant $J_{l,m}\in \mathbb{R}$. These coupling constants subsume the inverse temperature parameter *β* found in the single-group Curie-Weiss model (see e.g. [27, Chapters IV and V] for a thorough discussion of the classical Curie-Weiss model). In fact, in the special case of *M* = 1, the definition of ℍ reduces to the Hamiltonian of the Curie-Weiss model. We note that, depending on the signs of the coupling parameters *J*_*l*,*m*_, the value of ℍ varies with the voting configuration. In the present context of voting, we interpret $ℍ\left(x_{1},\ldots ,x_{N}\right)$ as a measure of conflict in society surrounding a particular issue which gives rise to votes $\left(x_{1},\ldots ,x_{N}\right)\in {\left\{-1,1\right\}}^{N}$. If all coupling parameters are positive, there are two configurations that have the lowest possible level of conflict: the unanimous configurations (−1,…,−1) and (1,…,1). All other configurations receive higher values $ℍ\left(x_{1},\ldots ,x_{N}\right)$. The highest levels are achieved when the votes are evenly split in each group (or closest to it in case of odd group sizes). We define the coupling matrix *J* to have entries equal to the constants above:

$$J:={\left(J_{l,m}\right)}_{l,m\in {\mathbb{N}}_{M}}\in {\mathbb{R}}^{M\times M}.$$ (1.3)

**Definition 1.3.** *Let A > 0 stand for the statement that $A\in {\mathbb{R}}^{M\times M}$ is a symmetric positive definite matrix, i.e. the Euclidean inner product ⟨Ax,x⟩ is positive for all $x\in {\mathbb{R}}^{M},x\neq 0$, and let A≥0 mean A is positive semi-definite, i.e. ⟨Ax,x⟩≥0 holds for all $x\in {\mathbb{R}}^{M}$.*

**Remark 1.4.** *We will assume J > 0 throughout this article. This assumption can be interpreted as the existence of stronger coupling between voters belonging to the same group versus voter coupling between groups. The assumption also allows for negative coupling between different groups, which models an antagonistic relationship between groups.*

**Definition 1.5.** *Let $J\in {\mathbb{R}}^{M\times M}$ with J > 0 and ℍ as defined in (2). The block spin Ising model’s probability measure ℙ, which gives the probability of each of the 2^N^ voting configurations, is defined by*

$$\mathbb{P}\left(X_{1}=x_{1},\ldots ,X_{N}=x_{N}\right):=Z^{-1}e^{-ℍ\left(x_{1},\ldots ,x_{N}\right)}$$ (1.4)

*for all $\left(x_{1},\ldots ,x_{N}\right)\in {\left\{-1,1\right\}}^{N}$, where Z is a normalisation constant which depends on N and J.*

### 1.2 Results

The model has three distinct regimes, in each of which the model behaves in a distinct fashion. This is reflected in the limiting distribution of the vector of suitably normalised sums of votes $\left(S_{1},\ldots ,S_{M}\right)$, where *S*_*l*_ is the sum of the votes in group $l\in {\mathbb{N}}_{M}$. This limiting distribution is distinct in each of the three regimes. These results can be found in several articles, e.g. [23,26,28]. The three regimes are called (in adaptation of the corresponding terms for the classical single-group Curie-Weiss model) the high temperature, the critical, and the low temperature regime. The high temperature regime is characterised by the difference between the identity matrix $I\in {\mathbb{R}}^{M\times M}$ and the coupling matrix *J* being a positive definite matrix, i.e. *I*–*J* > 0. The model is in the critical regime when I−J≥0 but I−J≯0. Finally, the low temperature regime is equivalent to I−J≱0. High temperature corresponds to high disorder, meaning the voters tend to have a mind of their own with weak dependence of the votes. Low temperature corresponds to strong couplings between votes. Thus the regime depends on the social cohesion present in a society. Culturally homogeneous societies would likely fall into the low temperature regime, whereas confederations with loosely related heterogeneous groups would be expected to fall into the high temperature regime assumption.

We will study the problem of detecting the *M* communities in the model in the high temperature and the low temperature regimes. In the latter case, we will make an assumption (see Definition 1.8) about the Hessian matrices at the minima of the function $F:{\mathbb{R}}^{M}\to \mathbb{R}$ defined by

$$F(x)=\frac{1}{2}x^{T}J^{-1}x-\sum_{l=1}^{M}\alpha_{l}\ln\cosh\left(\frac{x_{l}}{\sqrt{\alpha_{l}}}\right),\;x\in {\mathbb{R}}^{M},$$ (1.5)

which plays a crucial role in the analysis of the block spin Ising model. It appears in the de Finetti representation of the probability measure ℙ (see [26, Theorem 32] for more details). The de Finetti representation of the probabilities 1.4 as an integral over ${\mathbb{R}}^{M}$. The function *F* plays the role of exponentially weighting the points in the set ${\mathbb{R}}^{M}$. A smaller value of F(x) yields a higher weight in the integral. Thus, the minima of *F* are of particular importance, and their location depends on the regime the model is in.

**Definition 1.6.** *We will use the symbol C for positive constants which are independent of N but may depend on the coupling matrix J and the group size parameters $\alpha_{l}$. We make no claim as to the precise value of these constants, and in fact they may change from one line of a calculation to the next.*

**Theorem 1.7.** *In the high temperature regime, i.e. for I–J > 0, set H: = J^−1^−I. Then there is a positive constant $C_{high}$ such that for all N∈ℕ*


$$\left|𝔼\left(X_{i}X_{j}\right)-\frac{H_{\iota (i),\iota (j)}^{-1}}{N}\right|\leq C_{high}\frac{{\left(\ln N\right)}^{(6+M)/2}}{N^{2}},\;i,j\in {\mathbb{N}}_{N},i\neq j,$$

*holds, where we use the notation $H_{\iota (i),\iota (j)}^{-1}={\left(H^{-1}\right)}_{\iota (i),\iota (j)}.$*

This theorem is proved in Sect 3.

As in the high temperature regime, in the low temperature regime, i.e. I−J≱0, we address the non-critical case. Our definition of the low temperature non-critical case is similar to the corresponding definition for high temperature.

**Definition 1.8** (Low temperature non-critical case). *In the case that I−J≱0, we say that the model is in the low temperature regime. Moreover, if the Hessian of F at every point where the minimum is attained is positive definite, we say that the model is non-critical.*

**Lemma 1.9.** *In the low temperature non-critical case, the number of points where the minimum of F is attained is finite. We denote them by*

$${\left\{z^{\left(k\right)}\right\}}_{k\in {\mathbb{N}}_{K}},$$ (1.6)

*and the corresponding Hessian of F at the point z${\;}^{\left(\text{k}\right)}$ is denoted by H_k_ and is invertible for every $k\in {\mathbb{N}}_{K}$.*

Proof: Since F is dominated by the term $\frac{1}{2}x^{t}J^{-1}x$ for large ‖x‖, it follows that all points where the minimum is attained must be contained in a compact set $B\subset {\mathbb{R}}^{M}$ (we recall that J^−1^ is positive definite). Suppose that z is a point where the minimum is attained. The Hessian of F is positive definite at z by assumption. This implies that there is a neighbourhood $U\subset {\mathbb{R}}^{M}$ of z where F attains its minimum only at z. We obtain that the set of points where the minimum is reached consists of isolated points, and it is a closed set because F is continuous. This set cannot be infinite, otherwise it would contain an accumulation point of it since B is compact, and this accumulation point would not be isolated. □

We define

$${\vec{Z}}_{l}:=\frac{1}{\sqrt{\sum_{k}\frac{1}{\det(H_{k}{)}^{1/2}}}}(\begin{array}{c} \frac{1}{\det(H_{1}{)}^{1/4}}\tanh\left(z_{l}^{\left(1\right)}\right)\\ \frac{1}{\det(H_{2}{)}^{1/4}}\tanh\left(z_{l}^{\left(2\right)}\right)\\ \vdots \\ \frac{1}{\det(H_{K}{)}^{1/4}}\tanh\left(z_{l}^{\left(K\right)}\right) \end{array})\in {\mathbb{R}}^{K},\;l\in {\mathbb{N}}_{M}.$$ (1.7)

**Remark 1.10.** *Notice that the map*


$$y\in {\mathbb{R}}^{K}\mapsto \frac{1}{\sqrt{\sum_{k}\frac{1}{\det(H_{k}{)}^{1/2}}}}(\begin{array}{c} \frac{1}{\det(H_{1}{)}^{1/4}}\tanh\left(y_{1}\right)\\ \frac{1}{\det(H_{2}{)}^{1/4}}\tanh\left(y_{2}\right)\\ \vdots \\ \frac{1}{\det(H_{K}{)}^{1/4}}\tanh\left(y_{K}\right) \end{array})\in {\mathbb{R}}^{K}$$

*is bijective. We assume that there are no l and m with l≠m such that $z_{l}^{\left(k\right)}=z_{m}^{\left(k\right)}$ holds for every k=1,…,K. This is equivalent to assuming that*


$${\vec{Z}}_{l}\neq {\vec{Z}}_{m},\;l\neq m.$$

*The above implies that for every l≠m either $\left\|{\vec{Z}}_{l}\right\|\neq \left\|{\vec{Z}}_{m}\right\|$ or $\left\|{\vec{Z}}_{l}\right\|=\left\|{\vec{Z}}_{m}\right\|$ and $\left\langle {\vec{Z}}_{l},{\vec{Z}}_{m}\right\rangle <{\left\|{\vec{Z}}_{l}\right\|}^{2}={\left\|{\vec{Z}}_{m}\right\|}^{2}$, where the inequality derives from the non-collinearity of ${\vec{Z}}_{l}$ and ${\vec{Z}}_{m}$ and the equality condition in the Cauchy-Schwarz inequality. The vectors ${\vec{Z}}_{l}$ play a role in the group identification procedure in the high temperature regime, where it will allow us to separate the pair correlations belonging to different groups.*

**Theorem 1.11.** *Assume the model is in the low temperature regime and non-critical as per Definition 1.8, i.e. the Hessian H_k_ of F at z_k_ is positive definite for all k. Then the function F defined in (5) has a finite number of minima, $z^{\left(1\right)},\ldots ,z^{\left(K\right)}\in {\mathbb{R}}^{M}$. There is a positive constant $C_{low}$ such that for all N∈ℕ*


$$\left|𝔼\left(X_{i}X_{j}\right)-\left\langle {\vec{Z}}_{\iota (i)},{\vec{Z}}_{\iota (j)}\right\rangle \right|\leq C_{low}\frac{{\left(\ln N\right)}^{\left(M+3\right)/2}}{\sqrt{N}},\;i,j\in {\mathbb{N}}_{N},i\neq j.$$

This theorem is proved in Sect 3.

The main results of this article, the proof that the community detection problem has a solution with high probability and the algorithms for the reconstruction of the group structure of the model are found in Sects 2.2 and 2.3, respectively. Theorems 1.7 and 1.11 allow us to calculate approximations for the correlations $𝔼\left(X_{i}X_{j}\right)$ between votes for large *N*. We will use these approximations as a benchmark for the empirical correlations obtained from a sample of observations $x^{\left(1\right)},x^{\left(2\right)},\ldots ,x^{\left(n\right)}\in {\left\{-1,1\right\}}^{N}$ of voting configurations from the model:


$$\frac{1}{n}\sum_{t=1}^{n}x_{i}^{\left(t\right)}x_{j}^{\left(t\right)},\;i\neq j$$

(cf. Formulae (2.13) and (2.14)). From the asymptotic analysis of the block spin Ising model in Theorems 1.7 and 1.11, we know the large *N* value of $𝔼\left(X_{i}X_{j}\right)$, and by the law of large numbers the empirical correlations converge to these values as *n* goes to infinity. The empirical correlations also satisfy a large deviations principle (see Lemmas 2.7 and 2.21 and Proposition 2.2) which allows us to upper bound the probability of a significant deviation of the empirical correlations from these values by a function which exponentially decays to 0 with the number of observations *n*. Thus, with high probability, we obtain a sample of observations that are typical in that there are no large deviations of the empirical correlations from their expected values. Then we use an iterative algorithm to define an equivalence relation on the set of voters ${\mathbb{N}}_{N}$ that corresponds to the underlying group structure represented by *ι* which is assumed to be unknown to the observer of the voting configurations $x^{\left(1\right)},x^{\left(2\right)},\ldots ,x^{\left(n\right)}$. We next describe the algorithm informally for the high temperature regime (the low temperature regime algorithm is structurally similar). See the proofs of Theorems 1.12 and 1.13 for a rigorous description and Sect 1.3 for an example of the application of this algorithm.

We take a correlation


$$𝔼\left(X_{i^{*}}X_{j^{*}}\right)=\max_{i\neq j}\left\{𝔼\left(X_{i}X_{j}\right)\right\}$$

which according to Corollary 2.4, in the high temperature regime, corresponds to voters $i^{*},j^{*}\in {\mathbb{N}}_{N},i^{*}\neq j^{*}$, who both belong to the same group, namely group $l\in {\mathbb{N}}_{M}$ with the largest value $H_{l,l}^{-1}$ (cf. Theorem 1.7). We then identify the largest empirical correlations, which according to Lemma 2.21 belong to those voters *i* and *j* who indeed belong to said group, i.e. ι(i)=ι(j)=l. Having identified the voters belonging to group *l*, we remove from our set of empirical correlations all those elements that correspond to voter pairs which include at least one of the indices just identified as belonging to group *l*. Then we pick the group $l^{\prime }\in {\mathbb{N}}_{M}$ with the next largest value $H_{l^{\prime },l^{\prime }}^{-1}$, and repeat the last step. In each step, we identify at least one group of voters. Hence, the algorithm terminates after at most *M* steps. Thus, we provide an explicit algorithm of how to reconstruct the structure of the model.

We use the phrase ‘Property *f* holds with high probability as *n* goes to infinity’ in the sense that for any constant ε>0 there is a constant $n_{\varepsilon }\in \mathbb{N}$ such that for all $n\geq n_{\varepsilon }$ the probability that *f* holds is at least 1−ε.

The main theorems of this article are

**Theorem 1.12** (Group Identification and Reconstruction Procedure in the High Temperature Regime). *Let the model be in the high temperature regime. There is a fixed natural number $N_{\text{high}}$ and a constant δ > 0 (cf. Definition 2.5) such that if $N\geq N_{\text{high}}$ and n∈ℕ, a sample of n observations $x^{\left(1\right)},x^{\left(2\right)},\ldots ,x^{\left(n\right)}$ allows us to recover the group partition with high probability. The probability that we cannot recover the group partition is bounded above by the exponentially decaying function*


$$e_{high}(n):=N^{2}(n+1{)}^{2}e^{-\frac{1}{8}{\left(\frac{1}{8N}\delta \right)}^{2}n}.$$

and

**Theorem 1.13** (Group Identification and Reconstruction Procedure in the Low Temperature Regime). *Let the model be in the low temperature regime and non-critical. Let $z^{\left(1\right)},\ldots ,z^{\left(K\right)}\in {\mathbb{R}}^{M}$ be the minima of the function F, and assume there are no l and m with l≠m such that $z_{l}^{\left(k\right)}=z_{m}^{\left(k\right)}$ holds for every k=1,…,K. There is a fixed natural number $N_{\text{low}}$ and a constant γ > 0 (cf. Definition 2.19) such that if $N\geq N_{\text{low}}$ and n∈ℕ, a sample of n observations $x^{\left(1\right)},x^{\left(2\right)},\cdots ,x^{\left(n\right)}$ allows us to recover the group partition with high probability. The probability that we cannot recover it is bounded above by the following exponentially decaying function*


$$e_{\text{low}}(n):=N^{2}(n+1{)}^{2}e^{-\frac{1}{8}{\left(\frac{1}{8}\gamma \right)}^{2}n}.$$

**Remark 1.14.** *The upper bounds for the probabilities that the community detection problem has a solution are tight in the sense that Sanov’s Theorem provides a corresponding lower bound as well, and said lower bound features an exponential term*


$$e^{-\frac{1}{8}{\left(\frac{1}{8N}\delta^{\prime }\right)}^{2}n}\;and\;e^{-\frac{1}{8}{\left(\frac{1}{8}\gamma^{\prime }\right)}^{2}n},$$

*respectively, for some $\delta^{\prime }\geq \delta$ in the high temperature regime and some $\gamma^{\prime }\geq \gamma$ in the low temperature regime. As such, in the general case of M groups, it is not possible to improve these bounds.*

**Remark 1.15.** In the original articles [1,2] concerning the community detection problem in the block spin Ising model, the two groups considered where identical in all respects – group sizes and coupling constants. Therefore, community detection was only possible in terms of finding an equivalence relation on the set of voters ${\mathbb{N}}_{N}$ such as those constructed in the proofs of Theorems 1.12 and 1.13. For the more general situation of several groups of arbitrary sizes and coupling constants, we can go beyond this solution and identify the equivalence classes detected and specific groups of the model. One such case is when all groups are of different sizes, i.e. $N_{l}\neq N_{m}$ for all l≠m. Another example is $H_{l,l}^{-1}\neq H_{m,m}^{-1}$ for all l≠m. In fact, there is a way to explicitly identify the groups in most cases. Also, the algorithm allows identifying the group structure in case there is imperfect information about the structure of the model in terms of group sizes and coupling constants. An example of the reconstruction algorithm using simulated data which illustrates that the group sizes are not strictly necessary to reconstruct the group structure of the model is included as Supplementary Material to this article.

In the next subsection, we present an example for the application of the community detection algorithm to the problem of extremist political movements. The rest of this article consists of Sect 2 where we formally state and prove the community detection results, Sect 3 in which we prove Theorems 1.7 and 1.11, and finally Sect 4 which includes some results we use in the proofs in order to make the article as self-contained as possible.

### 1.3 Detection of the members of extremist political parties

In this section, we describe how our community detection algorithms can be employed to detect members of political movements or parties. In many countries, extremist political parties are forbidden by the authorities. Membership is usually prohibited and heavily penalised. As such, admitting to membership risks prosecution up to lengthy prison sentences. Even if an extremist political party is not prohibited, or else it is not a formal party but more of a loose movement or association of like-minded people, it may still be the case that members do not openly identify as such. Censure from mainstream segments of the population leads to the phenomenon of self-censoring and avoidance of stating their membership openly. Thus, if we have the task of quantifying the membership numbers of extremist parties in a certain population of *N* people, asking for their political affiliation directly may not lead to accurate numbers, as extremist affiliations will likely be underrepresented among the responses. Instead, we take the indirect route of applying a questionnaire with *n* questions with binary responses. These can be yes/no questions but also questions asking about two possible solutions to current political or societal problems.

Suppose there are two political parties in a country. Party 1 is an extremist political movement shunned by sympathisers of party 2, which is a mainstream moderate party, and ‘party 3’, the remainder of the population which is not politically engaged. We endeavour to assign each respondent correctly to one of the three parties. Suppose we have a sample $x^{\left(1\right)},\ldots ,x^{\left(n\right)}$ of *n* observations of voting configurations


$$x^{\left(t\right)}=\left(x_{1}^{\left(t\right)},\ldots ,x_{N}^{\left(t\right)}\right)\in {\left\{-1,1\right\}}^{N},\;t\in {\mathbb{N}}_{n},$$

obtained from the responses to our questionnaire for the entire population of N∈ℕ voters. The assumption about the population structure implies there are three groups in our block spin Ising model (cf. Definition 1.5). The coupling matrix $J={\left(J_{l,m}\right)}_{l,m\in {\mathbb{N}}_{3}}$ defined in (1.3) is assumed to be given by


$$J=\left(\begin{array}{ccc} 0.7 & -0.2 & 0.2\\ -0.2 & 0.6 & 0.1\\ 0.2 & 0.1 & 0.5 \end{array}\right),$$

which is positive definite. Moreover, it is easily checked that the matrix


$$I-J=\left(\begin{array}{ccc} 0.3 & 0.2 & -0.2\\ 0.2 & 0.4 & -0.1\\ -0.2 & -0.1 & 0.5 \end{array}\right)$$

is positive definite. Therefore, the model exhibits weak interactions between voters by virtue of being in the high temperature regime. We set *H*: = *J*^−1^−*I* (cf. Theorem 1.7), calculate


$$H^{-1}=\left(\begin{array}{ccc} 5.5517 & -2.7586 & 2.0690\\ -2.7586 & 2.7931 & -0.3448\\ 2.0690 & -0.3448 & 1.7586 \end{array}\right),$$

and order the diagonal entries of *H*^−1^:


$$H_{1,1}^{-1}>H_{2,2}^{-1}>H_{3,3}^{-1}.$$

We have


$$\eta :=\min_{l\neq m}\left\{\max\left\{H_{l.l}^{-1},H_{m,m}^{-1}\right\}-H_{l,m}^{-1}\right\}=3.1379,$$

$$\xi :=\min_{l\neq m}\left\{\left|H_{l.l}^{-1}-H_{m,m}^{-1}\right|\;|\;H_{l.l}^{-1}\neq H_{m,m}^{-1}\right\}=1.0345,$$

and set


δ:=min{η,ξ}=1.0345.

Next, we calculate the empirical correlations between two votes for each pair $i,j\in {\mathbb{N}}_{N}$, i≠j,


$${\bar{y}}_{i,j}:=\frac{1}{n}\sum_{t=1}^{n}x_{i}^{\left(t\right)}x_{j}^{\left(t\right)}.$$

Inspecting the empirical correlations, we notice that there is a cluster of ${\bar{y}}_{i,j}$ around the value $\frac{H_{1,1}^{-1}}{N}$, contained in the open interval $\left(\frac{H_{1,1}^{-1}}{N}-\frac{\delta }{2},\frac{H_{1,1}^{-1}}{N}+\frac{\delta }{2}\right)$. We find that this cluster is composed of $\frac{N_{1}\left(N_{1}-1\right)}{2}$ empirical correlations of the total $\frac{N\left(N-1\right)}{2}$ correlations corresponding to the population as a whole. Since $H_{1,1}^{-1}$ is the largest diagonal entry, the *N*_1_ indices *i* to be found in the cluster of ${\bar{y}}_{i,j}$ around the value $\frac{H_{1,1}^{-1}}{N}$ correspond to the members of group 1, i.e. members of the extremist political party. We now exclude all indices *i* belonging to extremists from our set of empirical correlations and thus obtain a reduced set of $\frac{\left(N_{2}+N_{3}\right)\left(N_{2}+N_{3}-1\right)}{2}$ of ${\bar{y}}_{i,j}$. We inspect this set of correlations and find that there is a cluster of ${\bar{y}}_{i,j}$ around the value $\frac{H_{2,2}^{-1}}{N}$, in an open interval $\left(\frac{H_{2,2}^{-1}}{N}-\frac{\delta }{2},\frac{H_{2,2}^{-1}}{N}+\frac{\delta }{2}\right)$, which contains $\frac{N_{2}\left(N_{2}-1\right)}{2}$ correlations. The *N*_2_ indices to be found in these correlations belong to members of the mainstream political party. We exclude all *N*_2_ of these indices from our correlations. The remaining $\frac{N_{3}\left(N_{3}-1\right)}{2}$ correlations featuring *N*_3_ indices belong to non-political members of the population. We note that the fortuitous clustering of the empirical correlations around the values $\frac{H_{l,l}^{-1}}{N}$ is, in fact, a high probability event, provided the sample size *n* is large enough. (Cf. Theorems 1.12 and 1.13.) This concludes the reconstruction of this model’s group structure from the voting data in the sample.

## 2 Exact recovery of the group structure

In this section we prove the two main Theorems 1.12 and 1.13 of this article. We will use the Theorems 1.7 and 1.11, which are proved in Sect 3.

### 2.1 Large deviations theory

Here we will define the probability space on which the samples of any possible size n∈ℕ generated by the model defined in Definition 1.5 live.

Let


Ξ:={−1,1}

be equipped with the *σ*-algebra ℱ of all subsets of Ξ. Let


$$\Omega_{1}:=\prod_{i=1}^{N}\Xi =\Xi^{{\mathbb{N}}_{N}}.$$

Moreover, we define


$$\Omega_{n}:=\prod_{i=1}^{n}\Omega_{1}=\Omega_{1}^{{\mathbb{N}}_{n}}\;n\in \mathbb{N},$$

provided with the *σ*-algebra ${ℱ}_{n}$ of all subsets. We set


$$\Omega :=\prod_{i=1}^{\infty }\Omega_{1}=\Omega_{1}^{\mathbb{N}},$$

with the *σ*-algebra ℱ generated by the cylinder sets of the from $𝒰\times \Omega_{1}^{\left\{n+1,n+2,\ldots \right\}}$, $𝒰\in \Omega_{n}$ and n∈ℕ.

We denote by


$$\omega ={\left\{\omega^{\left(t\right)}\right\}}_{t\in \mathbb{N}}$$

the elements of Ω. Here, $\omega^{\left(t\right)}\in \Omega_{1}$ for every *t*. We set the projections $X^{\left(t\right)}:\Omega \to \Omega_{1}$ given by


$$X^{\left(t\right)}(\omega )=\omega^{\left(t\right)}\in \Omega_{1},\;t\in \mathbb{N},$$

and


$$X_{i}^{\left(t\right)}(\omega )=\omega_{i}^{\left(t\right)}\in \Xi ,\;t\in \mathbb{N},i\in {\mathbb{N}}_{N}.$$

The random vector *X*${\;}^{\left(\text{t}\right)}$ corresponds to observation $t\in {\mathbb{N}}_{n}$. We define a product measure ν on (Ω,ℱ) using Kolmogorov’s extension theorem such that each *X*${\;}^{\left(\text{t}\right)}$ has a Curie-Weiss distribution according to Definition 1.5: for all $x\in \Omega_{1}$ and all t ∈ ℕ,


$$\nu \left(\left\{\omega \in \Omega \;|\;\omega^{\left(t\right)}=x\right\}\right)=Z^{-1}e^{-ℍ\left(x_{1},\ldots ,x_{N}\right)}.$$

We define the random variables


$$Y_{i,j}^{\left(t\right)}:=X_{i}^{\left(t\right)}X_{j}^{\left(t\right)},\;i,j\in {\mathbb{N}}_{N},i\neq j.$$

We will next define a probability measure $\mu_{i,j}$ on Ω under which, for fixed *i* and *j*, $Y_{i,j}^{\left(1\right)},Y_{i,j}^{\left(2\right)},\ldots$ are independent and identically distributed (i.i.d.) random variables, for fixed t∈ℕ, $Y_{i,j}^{\left(t\right)},Y_{i^{\prime },j^{\prime }}^{\left(t\right)},\ldots$ are in general neither independent nor identically distributed. We identify $X_{i}^{\left(1\right)}\equiv X_{i}$ with the random variables whose joint distribution is given by ℙ in Definition 1.5. First we define the probability measure $\mu_{i,j}:ℱ\to [0,1]$ by


$$\mu_{i,j}(\{r\}):=\nu \left(\left\{\omega \in \Omega \;|\;\omega_{i}^{\left(1\right)}\omega_{j}^{\left(1\right)}=r\right\}\right).$$

For every n∈ℕ, we define the product measure on ${ℱ}_{n}$ by


$$\mu_{i,j}^{n}:=\mu_{i,j}^{⊗(n)}.$$

Using Kolmogorov’s extension theorem, we define the measure $\mu_{i,j}$ on ℱ such that


$$\mu_{i,j}\left(𝒰\times \Omega_{1}^{\left\{n+1,n+2,\ldots \right\}}\right)=\mu_{i,j}^{n}(𝒰)$$

for all such cylinder sets $𝒰\times \Omega_{1}^{\left\{n+1,n+2,\ldots \right\}}$ described above.

We denote by $L_{n}^{Y_{i,j}}:ℱ\to [0,1]$ the empirical measure associated to the process ${\left(Y_{i,j}^{\left(t\right)}\right)}_{t\in \mathbb{N}}$, i.e.

$$L_{n}^{Y_{i,j}}(\{r\})=\frac{1}{n}\left|\{s\in {\mathbb{N}}_{n}\;|\;Y_{i,j}^{\left(s\right)}=r\}\right|,\;r\in \Xi .$$ (2.1)

We denote by ${ℳ}_{1}\left(\Xi \right)$ the set of probability measures on Ξ, equipped with the total variation topology. Let C(Ξ) be the set of real-valued functions on Ξ and we identify it with ${\mathbb{R}}^{2}$ using the map f↦(f(−1),f(1)). We provide C(Ξ) with the topology of ${\mathbb{R}}^{2}.$ We use the symbol I∈C(Ξ) for the identity I(r)=r,r∈Ξ, and for every f∈C(Ξ) and every $\nu \in {ℳ}_{1}\left(\Xi \right)$ we define


ν(f):=∫fdν.

Therefore, we identify every measure ν with a continuous real-valued function on C(Ξ). We set

$${\bar{Y}}_{i,j}^{\left(n\right)}:=\frac{1}{n}\sum_{t=1}^{n}Y_{i,j}^{\left(t\right)}=L_{n}^{Y_{i,j}}(I),$$ (2.2)

the average of $Y_{i,j}^{\left(t\right)}$ over all $t\in {\mathbb{N}}_{N}$. We set for every ε∈(0,1)

$${ℳ}_{1}\left(\Xi \right)⊃{𝒪}_{i,j}(\varepsilon ):=\{\nu \in {ℳ}_{1}\left(\Xi \right)\;|\;\left|\nu (I)-\mu_{i,j}(I)\right|\geq \varepsilon \},$$ (2.3)

which is a closed set that does not contain $\mu_{i,j}$. Note that $\mu_{i,j}(I)=𝔼\left(X_{\iota (i)}X_{\iota (j)}\right)$. We set


$$\Omega ⊃{𝒪}_{i,j}^{n}(\varepsilon ):=\left\{\omega \in \Omega \;|\;L_{n}^{Y_{i,j}(\omega )}\in {𝒪}_{i,j}(\varepsilon )\right\}.$$

For every two measures, $\nu ,\theta \in {ℳ}_{1}\left(\Xi \right),$ we denote by


$$H(\nu \;|\;\theta ):=\sum_{r\in \Omega_{1}}\nu (\{r\})\log\frac{\nu (\{r\})}{\theta (\{r\})}$$

the relative entropy of ν with respect to *θ*. Notice that this function $H\left(\cdot \;|\;\theta \right):{ℳ}_{1}\left(\Xi \right)\to \mathbb{R}\cup \left\{\infty \right\}$ is non-negative and concave as a function of ν, and *θ* is its only minimum [29, Definition 2.1.5 and the following remark].

**Lemma 2.1.** *Let ε∈(0,1) and suppose that $\nu ,\theta \in {ℳ}_{1}\left(\Xi \right)$ are such that*

|ν(I)−θ(I)|≥ε. (2.4)

*It follows that*

$$H(\nu \;|\;\theta )\geq \frac{1}{8}\varepsilon^{2}.$$ (2.5)

*Proof:* Set r=θ({−1}) and t=ν({−1}). Inequality (2.4) implies that

|−t+(1−t)−(−r+(1−r))|=2|t−r|≥ε. (2.6)

Moreover, we have that

$$f(t):=H(\nu \;|\;\theta )=t\log\left(\frac{t}{r}\right)+(1-t)\log\left(\frac{1-t}{1-r}\right).$$ (2.7)

As stated above, the minimum of H(ν|θ) as a function of ν is ν=θ. It follows that *f* attains its minimum at *r*, and f(r)=0. A first order Taylor series around *r* implies that

$$f(t)=\frac{1}{2}f^{\prime \prime }(\xi )(t-r{)}^{2}=\frac{1}{2}\left(\frac{1}{\xi }+\frac{1}{1-\xi }\right)(t-r{)}^{2}\geq \frac{1}{2}(t-r{)}^{2},$$ (2.8)

where ξ lies between *r* and *t*, and therefore ξ∈(0,1). Displays (2.6)-(2.8) imply the desired result. □

We need a very precise upper bound for the tail probabilities under $\mu_{i,j}$. Therefore, we will use results from the proof of Sanov’s Theorem to establish said upper bound instead of taking the statement from the theorem itself. The result given in the theorem is in terms of the lim sup of a sequence in the number of observations *n*, but we will use an upper bound to be found in (2.9) which allows us to establish precisely how large *n* has to be in order to have an upper bound on the probability of atypical observations.

**Proposition 2.2.** *For every n∈ℕ and every ε∈(0,1), we have the upper bound*


$$\mu_{i,j}\left({𝒪}_{i,j}^{n}(\varepsilon )\right)\leq (n+1{)}^{2}e^{-\frac{1}{8}\varepsilon^{2}n}.$$

*Proof:* The result follows by the proof of Sanov’s Theorem [29, Theorem 2.1.10]. We use Eq. (2.1.12) in [29] which in our case reads as the following equation (we denote by $P_{\mu_{i,j}}$ the probability induced by $\mu_{i,j}$):

$$P_{\mu_{i,j}}\left(\left\{L_{n}^{Y_{i,j}(\omega )}\in {𝒪}_{i,j}(\varepsilon )\right\}\right)=\mu_{i,j}\left({𝒪}_{i,j}^{n}(\varepsilon )\right)\leq (n+1{)}^{2}e^{-n\inf_{\eta \in {𝒪}_{i,j}(\varepsilon )\cup {ℒ}_{n}}H(\eta \;|\;\mu_{i,j})},$$ (2.9)

where ${ℒ}_{n}=\left\{\eta \;|\;\eta =L_{n}^{y},\;\text{for some }nandy\right\}$, see above Lemma 2.1.12 in [29]. The result follows by (2.9), the definition of ${𝒪}_{i,j}(\varepsilon )$ and Lemma 2.1, which implies


$$H(\eta |\mu_{i,j})\geq \frac{1}{8}\varepsilon^{2},$$

for every $\eta \in {𝒪}_{i,j}(\varepsilon ).$ □

### 2.2 High temperature group identification

**Lemma 2.3.** *Let A be a positive definite M×M matrix. Then*


$$A_{l,m}<\max\left\{A_{l,l},A_{m,m}\right\},\;l,m\in {\mathbb{N}}_{M},l\neq m$$

*holds.*

Proof: Let *e*_*l*_, $l\in {\mathbb{N}}_{M}$, stand for the l-th vector of the canonical basis of ${\mathbb{R}}^{M}$. Since A is positive definite, it has a square root *A*^1/2^ which is itself positive definite. We have for all l≠m


$$\langle Ae_{l},e_{m}\rangle =\langle A^{1/2}e_{l},A^{1/2}e_{m}\rangle <\left\|A^{1/2}e_{l}\right\|\left\|A^{1/2}e_{m}\right\|$$

$$=\langle Ae_{l},e_{l}\rangle^{1/2}\langle Ae_{m},e_{m}\rangle^{1/2}\leq {\left[\max\left\{\langle Ae_{l},e_{l}\rangle^{1/2},\langle Ae_{m},e_{m}\rangle^{1/2}\right\}\right]}^{2}.$$

The strict inequality above follows from the Cauchy-Schwarz inequality, since the invertibility of *A*^1/2^ implies $A^{1/2}e_{l}$ and $A^{1/2}e_{m}$ are not linearly dependent, and thus the inequality is strict. As $\langle Ae_{l},e_{m}\rangle =A_{l,m}$, we conclude that


$$A_{l,m}<\max\left\{A_{l,l},A_{m,m}\right\}.$$

□

Recall that *H*: = *J*^−1^−*I*>0 holds in the high temperature regime. The previous Lemma implies

**Corollary 2.4.** *We have for all $l,m\in {\mathbb{N}}_{M}$ with l≠m*


$$H_{l,m}^{-1}<\max\left\{H_{l,l}^{-1},H_{m,m}^{-1}\right\}.$$

Using this corollary, we define the constants δ > 0 and $N_{high}\in \mathbb{N}$, which are used in Theorem 1.12.

**Definition 2.5.** *Let*


$$\eta :=\min_{l\neq m}\left\{\max\left\{H_{l.l}^{-1},H_{m,m}^{-1}\right\}-H_{l,m}^{-1}\right\},$$

$$\xi :=\min_{l\neq m}\left\{\left|H_{l.l}^{-1}-H_{m,m}^{-1}\right|\;|\;H_{l.l}^{-1}\neq H_{m,m}^{-1}\right\}.$$

*We define*

δ:=min{η,ξ}, (2.10)

*which is a strictly positive constant by Corollary 2.4, and*

$${𝒪}^{n}:={⋃}_{i\neq j}{𝒪}_{i,j}^{n}\left(\frac{1}{8N}\delta \right).$$ (2.11)

*Moreover, we choose a fixed natural number $N_{\text{high}}$ satisfying (cf. Theorem 1.7)*

$$C_{high}\frac{(\ln N{)}^{(6+M)/2}}{N^{2}}\leq \frac{1}{8}\frac{1}{N}\delta ,$$ (2.12)

*for every $N\geq N_{\text{high}}$.*

**Remark 2.6.** Notice that Proposition 2.2 implies


$$\mathbb{P}\left(\left\{\omega \in {𝒪}^{n}\right\}\right)\leq \sum_{i\neq j}\mu_{i,j}\left({𝒪}_{i,j}^{n}\left(\frac{1}{8N}\delta \right)\right)\leq N^{2}(n+1{)}^{2}e^{-\frac{1}{8}{\left(\frac{1}{8N}\delta \right)}^{2}n}.$$

In the following, we use the standard notation in which a random sample (or an observation) is denoted by lowercase letters corresponding to the random variables represented by uppercase letters. More precisely, for a ω∈Ω, we denote by

$$x^{\left(t\right)}=X^{\left(t\right)}(\omega )=\omega^{\left(t\right)}\in \Omega_{1}={\left\{-1,1\right\}}^{N}.$$ (2.13)

We fix $\omega \notin {𝒪}^{n}$ for the rest of this section. Given *ω*, we obtain a sample $x^{\left(1\right)},x^{\left(2\right)},\ldots ,x^{\left(n\right)}$ and calculate the empirical correlations (the realisation of (2.2) given *ω*)

$${\bar{y}}_{i,j}:={\bar{y}}_{i,j}(\omega ):=\frac{1}{n}\sum_{t=1}^{n}x_{i}^{\left(t\right)}x_{j}^{\left(t\right)}$$ (2.14)

for all $i,j\in {\mathbb{N}}_{N}$ with i≠j.

**Lemma 2.7.** *Assume that $N\geq N_{\text{high}}$. Let n∈ℕ. Then we have*

$$\left|{\bar{y}}_{i,j}-\frac{H_{\iota (i),\iota (j)}^{-1}}{N}\right|\leq \frac{1}{4}\frac{1}{N}\delta \;i,j\in {\mathbb{N}}_{N},i\neq j.$$ (2.15)

*Proof:* Recall $L_{n}^{Y_{i,j}(\omega )}(I)={\bar{y}}_{i,j}$ and $\mu_{i,j}(I)=𝔼\left(X_{\iota (i)}X_{\iota (j)}\right)$. It follows that


$$\left|{\bar{y}}_{i,j}-𝔼\left(X_{\iota (i)}X_{\iota (j)}\right)\right|=\left|L_{n}^{Y_{i,j}(\omega )}(I)-\mu_{i,j}(I)\right|\leq \frac{1}{8}\frac{1}{N}\delta .$$

Theorem 1.7 and (2.12) imply


$$\left|𝔼\left(X_{\iota (i)}X_{\iota (j)}\right)-\frac{H_{\iota (i),\iota (j)}^{-1}}{N}\right|\leq \frac{1}{8}\frac{1}{N}\delta ,$$

so we obtain


$$\left|{\bar{y}}_{i,j}-\frac{H_{\iota (i),\iota (j)}^{-1}}{N}\right|\leq \frac{1}{4}\frac{1}{N}\delta .$$

□

Let $h_{\left(1\right)},h_{\left(2\right)},\ldots ,h_{\left(q\right)}$ be an enumeration of the set ${\left\{H_{l,l}^{-1}\right\}}_{l\in {\mathbb{N}}_{M}}$ with


$$h_{\left(1\right)}>h_{\left(2\right)}>\ldots >h_{\left(q\right)}.$$

We define a partition of the set of all groups ${\mathbb{N}}_{M}$ based on the values *h*_(*u*)_ above.

**Definition 2.8.** *Let $P_{1},\ldots ,P_{q}$ be a partition of ${\mathbb{N}}_{M}$ induced by*


$$l\in P_{u}\;⟺\;H_{l,l}^{-1}=h_{\left(u\right)},\;u\in {\mathbb{N}}_{q}.$$

Next we recursively define two set sequences which will be employed in the reconstruction algorithm of the group structure of the model.

**Definition 2.9.** *Let $A:=\left\{\left(i,j\right)\in {\mathbb{N}}_{N}\times {\mathbb{N}}_{N}\;|\;i\neq j\right\}$. We define*


$$G_{1}:=\left\{(i,j)\in A\;|\;\left|{\bar{y}}_{i,j}-\frac{h_{\left(1\right)}}{N}\right|\leq \frac{1}{4N}\delta \right\}$$

*and*


$$A_{1}:=\{(i,j)\in A\;|\;\exists r\text{ such that }(i,r)\in G_{1}\;or\;(r,j)\in G_{1}\}.$$

*Assume $G_{1},\ldots ,G_{u}$ and $A_{1},\ldots A_{u}$ have been defined for some 1≤u<q. Set*


$$G_{u+1}:=\left\{(i,j)\in A⧵A_{u}\;|\;\left|{\bar{y}}_{i,j}-\frac{h_{\left(u+1\right)}}{N}\right|\leq \frac{1}{4N}\delta \right\}$$

*and*


$$A_{u+1}:=\left\{(i,j)\in A\;|\;\exists v\leq u+1,\exists r\text{ such that }(i,r)\in G_{v}\;or\;(r,j)\in G_{v}\right\}.$$

**Remark 2.10.** *It is a direct consequence of Definition 2.9 that*


$$G_{u}\cap G_{v}=\emptyset ,\;u,v\in {\mathbb{N}}_{q},u\neq v,$$

$$A_{u}\subset A_{u+1},\;u\in {\mathbb{N}}_{q-1}.$$

In what follows, we will state and prove a series of lemmas, which taken together will allow us to construct an equivalence relation on ${\mathbb{N}}_{M}$ (see Proposition 2.17) based only on information contained in the sample of observations, and more specifically the empirical correlations ${\bar{y}}_{i,j}$. This equivalence relation corresponds to the partition into the sets $\iota^{-1}(l)$, $l\in {\mathbb{N}}_{M}$, and thus constitutes the solution to the community detection problem stated in Theorem 1.12.

**Lemma 2.11.** *Let (i,j)∈A. Then we have*


$$(i,j)\in G_{1}\;⟺\;\iota (i)=\iota (j)\in P_{1}.$$

*Proof:* Recall that we fixed $\omega \notin {𝒪}^{n}$ and hence ${\bar{y}}_{i,j}$ is fixed as well. Suppose $(i,j)\in G_{1}$. By Definition 2.9,


$$\left|{\bar{y}}_{i,j}-\frac{h_{\left(1\right)}}{N}\right|\leq \frac{1}{4N}\delta$$

holds. Lemma 2.7 supplies the upper bound


$$\left|{\bar{y}}_{i,j}-\frac{H_{\iota (i),\iota (j)}^{-1}}{N}\right|\leq \frac{1}{4N}\delta .$$

The two inequalities yield

$$\left|\frac{H_{\iota (i),\iota (j)}^{-1}}{N}-\frac{h_{\left(1\right)}}{N}\right|\leq \frac{1}{2N}\delta .$$ (2.16)

Note that $H_{k,l}^{-1}=h_{\left(1\right)}$ holds if and only if $k=l\in P_{1}$. By Definition 2.5,


$$H_{k,l}^{-1}\leq h_{\left(1\right)}-\delta ,\;k\neq l,$$

and


$$H_{k,k}^{-1}\leq h_{\left(1\right)}-\delta ,\;k\notin P_{1}.$$

The above and inequality (2.16) give


$$\iota (i)=\iota (j)\in P_{1}.$$

Thus, we have shown that $(i,j)\in G_{1}$ implies $\iota (i)=\iota (j)\in P_{1}$. The converse is proved analogously. □

**Lemma 2.12.** *Let $u\in {\mathbb{N}}_{q}$. Suppose we have*


$$(i,j)\in G_{v}\;⟺\;\iota (i)=\iota (j)\in P_{v}$$

*for all (i,j)∈A and all 1≤v<u. Then*


$$\iota (i)=\iota (j)\in P_{u}\;⟹\;(i,j)\in G_{u}$$

*holds for all (i,j)∈A.*

Proof: Let $\iota (i)=\iota (j)\in P_{u}$. To obtain a contradiction, suppose $\left(i,j\right)\in A_{u-1}$. Then there is some r≠i such that $\left(i,r\right)\in G_{v}$ or $\left(r,j\right)\in G_{v}$ for some v < u. Without loss of generality, suppose $\left(i,r\right)\in G_{v}$. By assumption, this implies $\iota (i)=\iota (r)\in P_{v}$. However, this contradicts $\iota (i)\in P_{u}$ because of $P_{v}\cap P_{u}=\emptyset$. Therefore, $\left(i,j\right)\notin A_{u-1}$ must hold. Since $\omega \notin {𝒪}^{n}$, Lemma 2.7 yields


$$\left|{\bar{y}}_{i,j}-\frac{h_{\left(u\right)}}{N}\right|\leq \frac{1}{4N}\delta ,$$

and thus $(i,j)\in G_{u}$ holds by definition of *G*_*u*_:


$$G_{u}:=\left\{(i,j)\in A⧵A_{u-1}\;|\;\left|{\bar{y}}_{i,j}-\frac{h_{\left(u\right)}}{N}\right|\leq \frac{1}{4N}\delta \right\}.$$

□

**Lemma 2.13.** *Let $u\in {\mathbb{N}}_{q}$. Suppose we have*


$$(i,j)\in G_{v}\;⟺\;\iota (i)=\iota (j)\in P_{v}$$

*for all (i,j)∈A and all 1≤v<u. Then*


$$(i,j)\in G_{u}\;⟹\;\iota (i),\iota (j)\notin P_{v},\;v<u.$$

*Proof:* Assume $(i,j)\in G_{u}$. By Definition 2.9, there is no $r\in {\mathbb{N}}_{N}$ such that

$$\left(i,r\right)\in G_{v}\;or\;\left(r,j\right)\in G_{v}$$ (2.17)

for some *v* < *u*, as the latter would imply $\left(i,j\right)\in A_{v}$ and thus $\left(i,j\right)\notin G_{u}$ in contradiction to our assumption.

To obtain a contradiction, assume that $\iota (i)\in P_{v}$ for some *v* < *u*. Since each group has at least two individuals (cf. Remark 1.2), there is an r≠i with $\iota (r)=\iota (i)\in P_{v}$. By assumption, this implies $(i,r)\in G_{v}$ as *v* < *u*. This contradicts (2.17), and hence we conclude that $\iota (i)\notin P_{v}$. $\iota (j)\notin P_{v}$ follows by the same arguments. □

**Lemma 2.14.** *Let $u\in {\mathbb{N}}_{q}$. Suppose we have*


$$(i,j)\in G_{v}\;⟺\;\iota (i)=\iota (j)\in P_{v}$$

*for all (i,j)∈A and all 1≤v<u. Then*


$$(i,j)\in G_{u}\;⟹\;\iota (i)=\iota (j)\in P_{u}$$

*holds for all (i,j)∈A.*

Proof: Let $(i,j)\in G_{u}$. By the last lemma, we have $\iota (i),\iota (j)\notin P_{v}$ for all v < u. As $(i,j)\in G_{u}$, we have

$$\left|{\bar{y}}_{i,j}-\frac{h_{\left(u\right)}}{N}\right|\leq \frac{1}{4N}\delta ,$$ (2.18)

To obtain a contradiction, assume ι(i)≠ι(j). Then we have


$$H_{\iota (i),\iota (j)}^{-1}\leq h_{\left(u\right)}-\delta$$

by Definition 2.5, which contradicts (2.18). Therefore, ι(i)=ι(j) must hold. Next assume $\iota (i)=\iota (j)\in P_{w}$ for some *w*>*u*. Once again, Definition 2.5 implies


$$H_{\iota (i),\iota (j)}^{-1}=h_{\left(w\right)}\leq h_{\left(u\right)}-\delta ,$$

and we have a contradiction to (2.18). Hence, $\iota (i)=\iota (j)\in P_{u}$ is proved. □

**Corollary 2.15.** *Let $u\in {\mathbb{N}}_{q}$. Suppose we have*


$$(i,j)\in G_{v}\;⟺\;\iota (i)=\iota (j)\in P_{v}$$

*for all (i,j)∈A and all 1≤v<u. Then*


$$\iota (i)=\iota (j)\in P_{u}\;⟺\;(i,j)\in G_{u}$$

*holds for all (i,j)∈A.*

*Proof:* The statement follows from Lemmas 2.12 and 2.14. □

**Proposition 2.16.** *For all (i,j)∈A and all $u\in {\mathbb{N}}_{q}$, we have*


$$\iota (i)=\iota (j)\in P_{u}\;⟺\;(i,j)\in G_{u}.$$

*Proof:* Together, Lemma 2.11 and Corollary 2.15 constitute a proof by induction over $u\in {\mathbb{N}}_{q}$ of the proposition’s statement. □

**Proposition 2.17.** *Let the relation ∼ on ${\mathbb{N}}_{N}$ be defined by*


$$i\sim j\;⟺\;i=j\;or\;\exists u\in {\mathbb{N}}_{q}:\;(i,j)\in G_{u},\;i,j\in {\mathbb{N}}_{N}.$$

*Then ∼ is an equivalence relation, and, for all $i,j\in {\mathbb{N}}_{N}$, i~j holds if and only if ι(i)=ι(j).*

*Proof:* By Proposition 2.16, for all (i,j)∈A, the existence of a $u\in {\mathbb{N}}_{q}$ such that $\left(i,j\right)\in G_{u}$ is equivalent to $\iota (i)=\iota (j)\in P_{u}$. The function $\iota :{\mathbb{N}}_{N}\to {\mathbb{N}}_{M}$ yields a partition of ${\mathbb{N}}_{N}$ with the equivalence classes given by $\iota^{-1}(l)$, $l\in {\mathbb{N}}_{M}$, i.e. the indices $i\in {\mathbb{N}}_{N}$ belonging to group *l*. Therefore, $\left(i,j\right)\in G_{u}$ is equivalent to the statement that *i* and *j* belong to the same equivalence class given by some group $l\in {\mathbb{N}}_{M}$. □

For the convenience of the reader, we restate Theorem 1.12. The theorem says that the problem of community detection has a solution which can be identified with high probability.

**Theorem 2.18** (Group Identification and Reconstruction Procedure in the High Temperature Regime). *Let δ and $N_{high}$ be as specified in Definition 2.5. Suppose that $N\geq N_{\text{high}}$ and n is a natural number. A sample of n observations $x^{\left(1\right)},x^{\left(2\right)},\ldots ,x^{\left(n\right)}$ allows us to recover the group partition of ${\mathbb{N}}_{N}$ with high probability. The probability that we cannot recover the group partition is bounded above by the exponentially decaying function*


$$e_{high}(n):=N^{2}(n+1{)}^{2}e^{-\frac{1}{8}{\left(\frac{1}{8N}\delta \right)}^{2}n}.$$

*Proof:* Recall the definition of ${𝒪}^{n}$ in (18) and that we have fixed $\omega \notin {𝒪}^{n}$. By Proposition 2.17, the set sequence *G*_*u*_, $u\in {\mathbb{N}}_{q}$, allows us to define an equivalence relation ∼ on ${\mathbb{N}}_{N}$ which corresponds to the group structure of the model in the sense that for all $i,j\in {\mathbb{N}}_{N}i\sim j$ holds if and only if *i* and *j* belong to the same group. Then the probability that recovery is impossible is bounded above by $\mathbb{P}\left(\left\{\omega \in {𝒪}^{n}\right\}\right)\leq e_{high}(n)$ according to Remark 2.6. This concludes the proof of Theorem 1.12. □

### 2.3 Low temperature group identification

For the next definition, recall (1.7):


$${\vec{Z}}_{l}:=\frac{1}{\sqrt{\sum_{k}\frac{1}{\det(H_{k}{)}^{1/2}}}}(\begin{array}{c} \frac{1}{\det(H_{1}{)}^{1/4}}\tanh\left(z_{l}^{\left(1\right)}\right)\\ \frac{1}{\det(H_{2}{)}^{1/4}}\tanh\left(z_{l}^{\left(2\right)}\right)\\ \vdots \\ \frac{1}{\det(H_{2}{)}^{1/4}}\tanh\left(z_{l}^{\left(K\right)}\right) \end{array})\in {\mathbb{R}}^{K},\;l\in {\mathbb{N}}_{M},$$

the notation ${\left\{z^{\left(k\right)}\right\}}_{k\in {\mathbb{N}}_{K}}$ for the minima of the function *F*, and Remark 1.10, and recall that we defined the subsets of Ω


$${𝒪}_{i,j}^{n}(\varepsilon ):=\left\{\omega \in \Omega \;|\;L_{n}^{Y_{i,j}(\omega )}\in {𝒪}_{i,j}(\varepsilon )\right\},\;\varepsilon >0.$$

**Definition 2.19.** *We set*


$$\eta :=\min\left\{\left|{\left\|{\vec{Z}}_{l}\right\|}^{2}-{\left\|{\vec{Z}}_{m}\right\|}^{2}\right|\;|\;\left\|{\vec{Z}}_{l}\right\|\neq \left\|{\vec{Z}}_{m}\right\|,l\neq m\right\},$$

$$\xi :=\min\left\{\left|{\left\|{\vec{Z}}_{l}\right\|}^{2}-\left\langle {\vec{Z}}_{l},{\vec{Z}}_{m}\right\rangle \right|\;|\;\left\|{\vec{Z}}_{l}\right\|=\left\|{\vec{Z}}_{m}\right\|,l\neq m\right\},$$

γ:=min{η,ξ}

*(note that γ is positive by Remark 1.10), and*

$${𝒰}^{n}:={⋃}_{i\neq j}{𝒪}_{i,j}^{n}\left(\frac{1}{8}\gamma \right).$$ (2.19)

*Moreover, we choose a fixed natural number $N_{\text{low}}$ satisfying (cf. Theorem 1.11)*

$$C_{low}\frac{(\ln N{)}^{(M+3)/2}}{N^{1/2}}\leq \frac{1}{8}\gamma$$ (2.20)

*for every $N\geq N_{\text{low}}$.*

**Remark 2.20.** *Notice that Proposition 2.2 implies that*


$$\mathbb{P}\left(\left\{\omega \in {𝒰}^{n}\right\}\right)\leq \sum_{i,j}\mu_{i,j}\left({𝒪}_{i,j}^{n}\left(\frac{1}{8}\gamma \right)\right)\leq N^{2}(n+1{)}^{2}e^{-\frac{1}{8}(\frac{1}{8}\gamma {)}^{2}n}.$$

Recall that Theorem 1.11 states


$$\left|𝔼\left(X_{i}X_{j}\right)-\left\langle {\vec{Z}}_{\iota (i)},{\vec{Z}}_{\iota (j)}\right\rangle \right|\leq C_{low}\frac{{\left(\ln N\right)}^{\left(M+3\right)/2}}{\sqrt{N}},\;i,j\in {\mathbb{N}}_{N},i\neq j.$$

We fix $\omega \notin {𝒰}^{n}$ for the rest of this section. Given *ω*, we obtain a sample $x^{\left(1\right)},x^{\left(2\right)},\ldots ,x^{\left(n\right)}$. Recall the definition of the empirical correlations


$${\bar{y}}_{i,j}:={\bar{y}}_{i,j}(\omega ):=\frac{1}{n}\sum_{t=1}^{n}x_{i}^{\left(t\right)}x_{j}^{\left(t\right)}$$

for all $i,j\in {\mathbb{N}}_{N}$ with i≠j.

**Lemma 2.21.** *Assume that $N\geq N_{\text{Low}}$. Set n∈ℕ. Then the following inequality holds:*

$$\left|{\bar{y}}_{i,j}-\left\langle {\vec{Z}}_{\iota (i)},{\vec{Z}}_{\iota (j)}\right\rangle \right|\leq \frac{1}{4}\gamma ,\;i\neq j.$$ (2.21)

*Proof:* Since $\omega \in ({𝒰}^{n}{)}^{c}$, it follows that (recall that $L_{n}^{Y_{i,j}(\omega )}(I)={\bar{y}}_{i,j}$ and $\mu_{i,j}(I)=𝔼(X_{\iota (i)}X_{\iota (j)})$ and (2.19), (2.3))

$$\left|L_{n}^{Y_{i,j}(\omega )}(I)-\mu_{i,j}(I)\right|=\left|{\bar{y}}_{i,j}-𝔼(X_{\iota (i)}X_{\iota (j)})\right|\leq \frac{1}{8}\gamma ,$$ (2.22)

for all i≠j. Recalling Theorem 1.11,


$$\left|𝔼(X_{i}X_{j})-\left\langle {\vec{Z}}_{\iota (i)},{\vec{Z}}_{\iota (j)}\right\rangle \right|\leq C_{low}\frac{{\left(\ln N\right)}^{\left(M+3\right)/2}}{\sqrt{N}}\leq \frac{1}{8}\gamma ,$$

we obtain

$$\left|{\bar{y}}_{i,j}-\left\langle {\vec{Z}}_{\iota (i)},{\vec{Z}}_{\iota (j)}\right\rangle \right|\leq \frac{1}{4}\gamma$$ (2.23)

for every i≠j. □

Let $g_{\left(1\right)},g_{\left(2\right)},\ldots ,g_{\left(q\right)}$ be an enumeration of the set ${\left\{{\left\|{\vec{Z}}_{l}\right\|}^{2}\right\}}_{l\in {\mathbb{N}}_{M}}$ with


$$g_{\left(1\right)}>g_{\left(2\right)}>\ldots >g_{\left(q\right)}.$$

We define a partition of the set of all groups ${\mathbb{N}}_{M}$ based on the values *g*_(*u*)_ above.

**Definition 2.22.** *Let $P_{1},\ldots ,P_{q}$ be a partition of ${\mathbb{N}}_{M}$ induced by*


$$l\in P_{u}\;⟺\;{\left\|{\vec{Z}}_{l}\right\|}^{2}=g_{\left(u\right)},\;u\in {\mathbb{N}}_{q}.$$

Next we recursively define two set sequences which will be employed in the reconstruction algorithm of the group structure of the model.

**Definition 2.23.** *Let $A:=\left\{\left(i,j\right)\in {\mathbb{N}}_{N}\times {\mathbb{N}}_{N}\;|\;i\neq j\right\}$. We define*


$$G_{1}:=\left\{(i,j)\in A\;|\;\left|{\bar{y}}_{i,j}-g_{\left(1\right)}\right|\leq \frac{1}{4}\gamma \right\}$$

*and*


$$A_{1}:=\{(i,j)\in A\;|\;\exists r\text{ such that }(i,r)\in G_{1}\;or\;(r,j)\in G_{1}\}.$$

*Assume $G_{1},\ldots ,G_{u}$ and $A_{1},\ldots A_{u}$ have been defined for some 1≤u<q. Set*


$$G_{u+1}:=\left\{(i,j)\in A⧵A_{u}\;|\;\left|{\bar{y}}_{i,j}-g_{\left(u+1\right)}\right|\leq \frac{1}{4}\gamma \right\}$$

*and*


$$A_{u+1}:=\left\{(i,j)\in A\;|\;\exists v\leq u+1,\exists r\text{ such that }(i,r)\in G_{v}\;or\;(r,j)\in G_{v}\right\}.$$

**Remark 2.24.** *It is a direct consequence of Definition 2.23 that*


$$G_{u}\cap G_{v}=\emptyset ,\;u,v\in {\mathbb{N}}_{q},u\neq v,$$

$$A_{u}\subset A_{u+1},\;u\in {\mathbb{N}}_{q-1}.$$

The proof of the next proposition follows the same steps as that of Proposition 2.17, using Lemma 2.21 instead of Lemma 2.7.

**Proposition 2.25.** *Let the relation ∼ on ${\mathbb{N}}_{N}$ be defined by*


$$i\sim j\;⟺\;i=j\;or\;\exists u\in {\mathbb{N}}_{q}:\;(i,j)\in G_{u},\;i,j\in {\mathbb{N}}_{N}.$$

*Then ∼ is an equivalence relation, and, for all $i,j\in {\mathbb{N}}_{N}$, i~j holds if and only if ι(i)=ι(j).*

**Theorem 2.26** (Group Identification and Reconstruction Procedure in the Low Temperature Regime). *Let the model be in the low temperature regime and non-critical. Let $z^{\left(1\right)},\ldots ,z^{\left(K\right)}\in {\mathbb{R}}^{M}$ be the minima of the function F, and assume there are no l and m with l≠m such that $z_{l}^{\left(k\right)}=z_{m}^{\left(k\right)}$ holds for every k=1,…,K. There is a fixed natural number $N_{\text{low}}$ and a constant γ > 0 (cf. Definition 2.19) such that if $N\geq N_{\text{low}}$ and n∈ℕ, a sample of n observations $x^{\left(1\right)},x^{\left(2\right)},\ldots ,x^{\left(n\right)}$ allows us to recover the group partition with high probability. The probability that we cannot recover it is bounded above by the exponentially decaying function*


$$e_{\text{low}}(n):=N^{2}(n+1{)}^{2}e^{-\frac{1}{8}{\left(\frac{1}{8}\gamma \right)}^{2}n}.$$

*Proof:* Recall the definition of ${𝒰}^{n}$ in (2.19) and that we have fixed $\omega \notin {𝒰}^{n}$. By Proposition 2.25, the set sequence *G*_*u*_, $u\in {\mathbb{N}}_{q}$, allows us to define an equivalence relation ∼ on ${\mathbb{N}}_{N}$ which corresponds to the group structure of the model in the sense that for all $i,j\in {\mathbb{N}}_{N}i\sim j$ holds if and only if *i* and *j* belong to the same group. Then the probability that recovery is impossible is bounded above by $\mathbb{P}\left(\left\{\omega \in {𝒰}^{n}\right\}\right)\leq e_{\text{low}}(n)$ according to Remark 2.20. This concludes the proof of Theorem 1.13. □

## 3 Proofs of Theorems 1.7. and 1.11

Recall that


$$F(x)=\frac{1}{2}x^{T}J^{-1}x-\sum_{l=1}^{M}\alpha_{l}\ln\cosh\left(\frac{x_{l}}{\sqrt{\alpha_{l}}}\right),\;x\in {\mathbb{R}}^{M}.$$

We define for fixed $i,j\in {\mathbb{N}}_{N}$ with i≠j

$$Z_{2}(N):=Z_{2}(N;i,j):=\int_{{\mathbb{R}}^{M}}e^{-NF(x)}\tanh\left(\frac{x_{\iota (i)}}{\sqrt{\alpha_{\iota (i)}}}\right)\tanh\left(\frac{x_{\iota (j)}}{\sqrt{\alpha_{\iota (j)}}}\right)dx$$ (3.1)

and

$$Z_{0}(N):=\int_{{\mathbb{R}}^{M}}e^{-NF(x)}dx.$$ (3.2)

Then the representation

$$𝔼\left(X_{i}X_{j}\right)=\frac{Z_{2}(N)}{Z_{0}(N)}$$ (3.3)

holds by Theorem 4.1 in the Appendix.

### 3.1 Proof of Theorem 1.7

**Lemma 3.1.** *In the high temperature regime, where H = J^−1^−I>0, 0 is the only minimum of F and the lower bound*


$$F(x)\geq \frac{1}{2}\langle Hx,x\rangle \geq c_{H}{\left\|x\right\|}^{2},$$

*holds, where c_H_>0 is half of the minimum eigenvalue of H*.

*Proof:* Let ${𝐇}_{F}:{\mathbb{R}}^{M}\to \mathbb{R}$ be the Hessian matrix of *F* at $x\in {\mathbb{R}}^{M}$. A direct calculation using hyperbolic trigonometric identities shows that


$$({𝐇}_{F}{)}_{l,m}(x)=H_{l,m}+\delta_{l,m}\tanh^{2}\left(\frac{x_{l}}{\sqrt{\alpha_{l}}}\right),$$

where $\delta_{l,m}$ is the Kronecker delta. This implies that ${𝐇}_{F}(x)$ is positive definite for all $x\in {\mathbb{R}}^{M}$. Since $F^{\prime }(0)=0$, 0 is the only minimum of *F*, and, due to F(0)=0, *F* is strictly positive on the complement of {0}. Moreover, a Taylor series expansion of the function [0,1]∋t↦F(tx) in *t* of order 1 with remainder of order 2 in integral form shows that


$$F(x)=\int_{0}^{1}\langle ({𝐇}_{F}{)}_{i,j}(tx)x,x\rangle (1-t)\;dt\geq \frac{1}{2}\langle Hx,x\rangle \geq c_{H}{\left\|x\right\|}^{2},$$

where in the above equation we used $F^{\prime }(0)=0$ and the spectral theorem which yields strict positivity of the at most *M* eigenvalues of the self-adjoint *H*. □

We restate Theorem 1.7 for the convenience of the reader:

**Theorem 3.2.** *In the high temperature regime, i.e. for I–J > 0, set H: = J^−1^−I. Then there is a positive constant $C_{high}$ such that for all N∈ℕ*


$$\left|𝔼\left(X_{i}X_{j}\right)-\frac{H_{\iota (i),\iota (j)}^{-1}}{N}\right|\leq C_{high}\frac{{\left(\ln N\right)}^{(6+M)/2}}{N^{2}}\;i,j\in {\mathbb{N}}_{N},i\neq j,$$

*holds, where we use the notation $H_{\iota (i),\iota (j)}^{-1}={\left(H^{-1}\right)}_{\iota (i),\iota (j)}.$*

*Proof:* We will show the statement for ι(i)=ι(j). The case ι(i)≠ι(j) can be handled analogously. For any set $A\subset {\mathbb{R}}^{M}$, let $A^{c}:={\mathbb{R}}^{M}\backslash A$ be the complement. We will work with *Z*_2_(*N*) and *Z*_0_(*N*) defined in (3.1) and (3.2). We denote by


$$B(z,r):=\left\{x\in {\mathbb{R}}^{M}\;|\;\|x-z\|<r\right\},$$

the ball with radius *r*, centred at *z*, and we set (see Lemma 3.1)


$$r_{N}:=\sqrt{\frac{\left(M+4)\ln(N\right)}{c_{H}(N-1)}},$$

which is selected in order to fulfil $e^{-(N-1)c_{H}r_{N}^{2}}\leq \frac{1}{N^{M+4}}$. We split *Z*_2_(*N*) and *Z*_0_(*N*) into integrals over $B\left(0,r_{N}\right)$ and over $B(0,r_{N}{)}^{c}$. Lemma 3.1 implies that


$$\int_{B(0,r_{N}{)}^{c}}e^{-NF(x)}\tanh^{2}\left(\frac{x_{\iota (i)}}{\sqrt{\alpha_{\iota (i)}}}\right)\;dx$$

$$\leq e^{-\left(N-1\right)c_{H}r_{N}^{2}}\int_{B(0,r_{N}{)}^{c}}e^{-F(x)}\tanh^{2}\left(\frac{x_{\iota (i)}}{\sqrt{\alpha_{\iota (i)}}}\right)\;dx$$

$$\leq \frac{1}{N^{M+4}}\int_{{\mathbb{R}}^{M}}e^{-c_{H}{\left\|x\right\|}^{2}}\tanh^{2}\left(\frac{x_{\iota (i)}}{\sqrt{\alpha_{\iota (i)}}}\right)\;dx\leq C\frac{1}{N^{M+4}},$$ (3.4)

and likewise we conclude that

$$\int_{B(r_{N},0{)}^{c}}e^{-NF(x)}\;dx\leq C\frac{1}{N^{M+4}}$$ (3.5)

and

$$\int_{B(0,r_{N}{)}^{c}}e^{-N\langle Hx,x\rangle }\frac{x_{\iota (i)}^{2}}{\alpha_{\iota (i)}}\;dx\leq C\frac{1}{N^{M+4}},\;\int_{B(0,r_{N}{)}^{c}}e^{-N\langle Hx,x\rangle }\;dx\leq C\frac{1}{N^{M+4}}.$$ (3.6)

Notice that $\frac{d}{ds}\ln\cosh(s)=\tanh(s)$ and $\frac{d}{ds}\tanh(s)=1-\tanh^{2}(s)$. This implies that all even derivatives of lncosh(s) are polynomials with even powers of tanh(s) and all odd derivatives of lncosh(s) are polynomials with odd powers of tanh(s). Therefore, all such derivatives are uniformly bounded on ℝ, and odd derivatives evaluated at 0 vanish (and the same holds for *F*). Taking this into consideration, and using that F(0)=0 and the Hessian of *F* at 0 is *H*, the remainder formula in Taylor’s Theorem leads to

$$\left|F(x)-\frac{1}{2}\langle Hx,x\rangle \right|\leq C{\left\|x\right\|}^{4},$$ (3.7)

for some constant *C*, independent of *x* (for ‖x‖≤1 we use Taylor’s Theorem, for ‖x‖>1 we use that *F*(*x*) and ⟨Hx,x⟩ grow quadratically in ‖x‖). Note that for positive *s* and *t*, s≤t, the mean value theorem implies that $\left|e^{-s}-e^{-t}\right|=\left|e^{-s}\right|\left|e^{s-t}-1\right|\leq e^{-s}|s-t|\leq |s-t|$. This, together with the fact that tanh is bounded and (3.7), implies that

$$|\int_{B(0,r_{N})}e^{-NF(x)}\tanh^{2}\left(\frac{x_{\iota (i)}}{\sqrt{\alpha_{\iota (i)}}}\right)\;dx-\int_{B(0,r_{N})}e^{-N\frac{1}{2}\langle Hx,x\rangle }\tanh^{2}\left(\frac{x_{\iota (i)}}{\sqrt{\alpha_{\iota (i)}}}\right)\;dx|\;\leq CN\int_{B(0,r_{N})}\tanh^{2}\left(\frac{x_{\iota (i)}}{\sqrt{\alpha_{\iota (i)}}}\right){\left\|x\right\|}^{4}\;dx\;\leq CN\int_{B(0,r_{N})}{\left\|x\right\|}^{6}\;dx\;\leq CNr_{N}^{6+M}\leq C{\left(\ln N\right)}^{(6+M)/2}\frac{1}{N^{(4+M)/2}}.$$ (3.8)

Likewise, we get

$$|\int_{B(0,r_{N})}e^{-NF(x)}dx-\int_{B(0,r_{N})}e^{-N\frac{1}{2}\langle Hx,x\rangle }dx|\leq C{\left(\ln N\right)}^{(4+M)/2}\frac{1}{N^{(2+M)/2}}.$$ (3.9)

The Taylor series of tanh at 0 (up to second order, using that $\tanh(0)=0=\tanh^{\prime \prime }(0)$ and that $\tanh(0)=0=\tanh^{\prime \prime }(0)$ is uniformly bounded), implies that there is a constant *C* such that


$$|\tanh(s)-s|\leq C|s{|}^{3},$$

and this leads to

$$\;|\int_{B(0,r_{N})}e^{-N\frac{1}{2}\langle Hx,x\rangle }\tanh^{2}\left(\frac{x_{\iota (i)}}{\sqrt{\alpha_{\iota (i)}}}\right)\;dx-\int_{B(0,r_{N})}e^{-N\frac{1}{2}\langle Hx,x\rangle }\frac{x_{\iota (i)}^{2}}{\alpha_{\iota (i)}}\;dx|\;\leq C\int_{B(0,r_{N})}{\left\|x\right\|}^{4}\;dx\leq CNr_{N}^{4+M}\leq C{\left(\ln N\right)}^{(4+M)/2}\frac{1}{N^{(4+M)/2}}.$$ (3.10)

Next, Proposition 4.2 implies that

$$\int_{{\mathbb{R}}^{M}}e^{-N\frac{1}{2}\langle Hx,x\rangle }\frac{x_{\iota (i)}^{2}}{\alpha_{\iota (i)}}\;dx=\frac{(2\pi {)}^{M/2}}{\det(H{)}^{1/2}}\frac{1}{N^{M/2+1}}H_{\lambda ,\lambda }^{-1},\;\;\int_{{\mathbb{R}}^{M}}e^{-N\frac{1}{2}\langle Hx,x\rangle }\;dx=\frac{(2\pi {)}^{M/2}}{\det(H{)}^{1/2}}\frac{1}{N^{M/2}}.$$ (3.11)

It follows from (3.1), (3.4), (3.6),(3.8), (3.10), and (3.11) that

$$|Z_{2}(N)-\frac{(2\pi {)}^{M/2}}{\det(H{)}^{1/2}}\frac{1}{N^{M/2+1}}H_{\lambda ,\lambda }^{-1}|\leq C\ln(N{)}^{(6+M)/2}\frac{1}{N^{(4+M)/2}}$$ (3.12)

and it follows from (3.2), (3.5), (3.6), (3.9), and (3.11) that

$$|Z_{0}(N)-\frac{(2\pi {)}^{M/2}}{\det(H{)}^{1/2}}\frac{1}{N^{M/2}}|\leq C\ln(N{)}^{(4+M)/2}\frac{1}{N^{(2+M)/2}}.$$ (3.13)

Set


$$A:=\frac{(2\pi {)}^{M/2}}{\det(H{)}^{1/2}}\frac{1}{N^{M/2+1}}H_{\lambda ,\lambda }^{-1},\;B:=\frac{(2\pi {)}^{M/2}}{\det(H{)}^{1/2}}\frac{1}{N^{M/2}}.$$

Then, using (3.12) and (3.13), we obtain

$$\left|\frac{Z_{2}(N)}{Z_{0}(N)}-\frac{1}{N}H_{\lambda ,\lambda }^{-1}\right|=\left|\frac{Z_{2}(N)-A}{Z_{0}(N)}+A\left(\frac{1}{Z_{0}}-\frac{1}{B}\right)\right|\;\leq \left|\frac{Z_{2}(N)-A}{Z_{0}(N)}\right|+|A|\frac{\left|Z_{0}(N)-B\right|}{\left|Z_{0}(N)||B\right|}\leq C\ln(N{)}^{(6+M)/2}\frac{1}{N^{2}}.$$ (3.14)

Displays (3.3) and (3.14) lead to the desired result. □

### 3.2 Proof of Theorem 1.11

Recall the definition of *F* given in (1.5) and Lemma 1.9.

**Lemma 3.3.** *There exist constants δ>0 and c > 0 such that the following estimation holds:*

$$F(x)-\alpha \geq c{\left\|x-z^{\left(k\right)}\right\|}^{2},\;\text{for}\;\left\|x-z^{\left(j\right)}\right\|\geq \delta ,j\neq k,$$ (3.15)

*where α is the minimum value of F, and the balls $B\left(z^{\left(k\right)},\delta \right)$ are disjoint for k∈{1,…,K}. Also, for every k and every $y\;\in \;{\mathbb{R}}^{M}$,*

$$\langle H_{k}y,y\rangle \geq c\|y{\|}^{2}.$$ (3.16)

*Proof:* The continuity of the Hessian of *F* shows that there are small enough numbers ε>0 and δ>0 such that $H_{F}-\varepsilon$ is positive in $B\left(z^{\left(k\right)},\delta \right)$ for every *k* (and we take *δ* small enough that these balls are disjoint). A first order Taylor series expansion (with second order remainder in the integral form) for the function $[0,1]∋t\to F\left(tx+(1-t)z^{\left(k\right)}\right)$ shows that

$$F(x)=\int_{0}^{1}\langle ({𝐇}_{F}{)}_{i,j}\left(tx+(1-t)z^{\left(k\right)}\right)\left(x-z^{\left(k\right)}\right),\left(x-z^{\left(k\right)}\right)\rangle (1-t)\;dt\;\geq \frac{1}{2}\left\langle \varepsilon \left(x-z^{\left(k\right)}\right),\left(x-z^{\left(k\right)}\right)\right\rangle ,$$ (3.17)

for every $x\in B\left(z^{\left(k\right)},\delta \right)$. For large enough ‖x‖, *F*(*x*) is dominated by $\frac{1}{2}x^{T}J^{-1}x\geq c\|x{\|}^{2}$, where *c* > 0 is any constant smaller than the smallest eigenvalue of *J*^−1^. For large enough *x*, there is a constant *c* such that $\|x{\|}^{2}\geq c\|x-z^{\left(k\right)}{\|}^{2}$. We conclude that there is a constant *c* > 0 such that

$$F(x)\geq c{\left\|x-z^{\left(k\right)}\right\|}^{2}$$ (3.18)

for large enough *x* and every *k*. Eqs (3.17) and (3.18) imply (3.15) for every *x* in the complement of a compact set 𝒞 not intersecting $B\left(z^{\left(k\right)},\delta \right)$, for every *k*. On 𝒞, F−α attains its minimum, which we call β>0 (it cannot be smaller or equal 0 because *F* attains its minimum only at the points *z*${\;}^{\left(\text{k}\right)}$). On the set 𝒞, (3.15) holds true as well. This is verified with the following estimation:

$$F(x)-\alpha \geq \beta \geq \beta \frac{1}{\max_{x\in 𝒞}(\|x-z_{k}{\|}^{2})}{\left\|x-z^{\left(k\right)}\right\|}^{2}$$ (3.19)

for every x∈𝒞.

We restate Theorem 1.11 for the reader’s convenience: □

**Theorem 3.4.** *Assume the model is in the low temperature regime and non-critical as per Definition 1.8, i.e. the Hessian H_k_ of F at z_k_ is positive definite for all k. Then the function F defined in (1.5) has a finite number of minima, $z^{\left(1\right)},\ldots ,z^{\left(K\right)}\in {\mathbb{R}}^{M}$. There is a positive constant $C_{low}$ such that for all N∈ℕ*


$$\left|𝔼\left(X_{i}X_{j}\right)-\left\langle {\vec{Z}}_{\iota (i)},{\vec{Z}}_{\iota (j)}\right\rangle \right|\leq C_{low}\frac{{\left(\ln N\right)}^{\left(M+3\right)/2}}{\sqrt{N}},\;i,j\in {\mathbb{N}}_{N},i\neq j.$$

Recall the definitions of *Z*_2_(*N*), and *Z*_0_(*N*) in (3.1) and (3.2), as well as the expression


$$𝔼\left(X_{i}X_{j}\right)=\frac{Z_{2}(N)}{Z_{0}(N)}$$

given in Theorem 4.1.

We set (cf. Lemma 3.3)


$$\rho_{N}:=\sqrt{\frac{\left(M+3)\ln(N\right)}{c(N-1)}},$$

which is selected in order to fulfil $e^{-(N-1)c\rho_{N}^{2}}\leq \frac{1}{N^{M+3}}$ (and we assume that *N* is large enough such that $\rho_{N}<\delta$). Lemma 3.3 implies that

$$\int_{({⋃}_{k=1}^{K}B(z_{k},\rho_{N}){)}^{c}}e^{-N(F(x)-\alpha )}\tanh^{2}\left(\frac{x_{\iota (i)}}{\sqrt{\alpha_{\iota (i)}}}\right)\;dx\;\leq \sum_{k=1}^{K}\int_{(B(z_{k},\delta )⧵B(z_{k},\rho_{N}))\cup ({⋃}_{j=1}^{K}B(z_{j},\delta ){)}^{c}}e^{-N(F(x)-\alpha )}\tanh^{2}\left(\frac{x_{\iota (i)}}{\sqrt{\alpha_{\iota (i)}}}\right)\;dx\;\leq \sum_{k=1}^{K}e^{-\left(N-1\right)c\rho_{N}^{2}}\int_{{\mathbb{R}}^{M}}e^{-c{\left\|x-z^{\left(k\right)}\right\|}^{2}}\tanh^{2}\left(\frac{x_{\iota (i)}}{\sqrt{\alpha_{\iota (i)}}}\right)\;dx\;\leq \frac{1}{N^{M+3}}\sum_{k=1}^{K}\int_{{\mathbb{R}}^{M}}e^{-c{\left\|x-z^{\left(k\right)}\right\|}^{2}}\tanh^{2}\left(\frac{x_{\iota (i)}}{\sqrt{\alpha_{\iota (i)}}}\right)\;dx\leq C\frac{1}{N^{M+3}},$$ (3.20)

and likewise we conclude that

$$\int_{({⋃}_{k=1}^{K}B(z_{k},\rho_{N}){)}^{c}}e^{-N(F(x)-\alpha )}\;dx\leq C\frac{1}{N^{M+3}}$$ (3.21)

and (see (3.16))

$$\sum_{k=1}^{K}\int_{B(z_{k},\rho_{N}{)}^{c}}e^{-\frac{N}{2}\langle H_{k}(x-z^{\left(k\right)}),(x-z^{\left(k\right)})\rangle }\left(\|x{\|}^{2}+1\right)\;dx\leq C\frac{1}{N^{M+3}}.$$ (3.22)

Notice that $\frac{d}{ds}\ln\cosh(s)=\tanh(s)$ and $\frac{d}{ds}\tanh(s)=1-\tanh^{2}(s)$. This implies that all even derivatives of lncosh(s) are polynomials with even powers of tanh(s) and all odd derivatives of lncosh(s) are polynomials with odd powers of tanh(s). Therefore, all such derivatives are uniformly bounded in ℝ. Next, we recall that $F\left(z^{\left(k\right)}\right)=\alpha$ and the Hessian of *F* at *z*${\;}^{\left(\text{k}\right)}$ is *H*_*k*_. A second order Taylor series expansion (with third order remainder in the mean value form) for the function $[0,1]∋t\to F\left(tx+(1-t)z^{\left(k\right)}\right)$ shows that, for every *k*,

$$\left|F(x)-\alpha -\frac{1}{2}\left\langle H_{k}\left(x-z^{\left(k\right)}\right),x-z^{\left(k\right)}\right\rangle \right|\leq C{\left\|x-z^{\left(k\right)}\right\|}^{3},$$ (3.23)

on $B\left(z^{\left(k\right)},\delta \right)$. Notice that for positive *s* and *t* with s≤t we have that the mean value theorem implies that $\left|e^{-s}-e^{-t}\right|=\left|e^{-s}\right|\left|e^{s-t}-1\right|\leq e^{-s}|s-t|\leq |s-t|$. This, together with the fact that tanh is bounded and (3.23), implies that

$$\;|\;\int_{B\left(z^{\left(k\right)},\rho_{N}\right)}e^{-N(F(x)-\alpha )}\tanh^{2}\left(\frac{x_{\iota (i)}}{\sqrt{\alpha_{\iota (i)}}}\right)\;dx\;\;-\int_{B(\rho_{N},z_{k})}e^{-\frac{N}{2}\langle H_{k}(x-z^{\left(k\right)}),(x-z^{\left(k\right)})\rangle }\tanh^{2}\left(\frac{x_{\iota (i)}}{\sqrt{\alpha_{\iota (i)}}}\right)\;dx\;|\;\leq CN\int_{B\left(z^{\left(k\right)},\rho_{N}\right)}{\left|x-z^{\left(k\right)}\right|}^{3}\;dx\leq CNr_{N}^{3+M}\;\leq CN{\left(\ln N\right)}^{(3+M)/2}\frac{1}{N^{(3+M)/2}}\leq C{\left(\ln N\right)}^{(3+M)/2}\frac{1}{N^{(1+M)/2}}$$ (3.24)

Likewise, we get

$$\;\left|\;\int_{B\left(z^{\left(k\right)},\rho_{N}\right)}e^{-N(F(x)-\alpha )}\;dx-\int_{B\left(z^{\left(k\right)},\rho_{N}\right)}e^{-\frac{N}{2}\langle H_{k}(x-z^{\left(k\right)}),(x-z^{\left(k\right)})\rangle }\;dx\;\right|\;\leq C{\left(\ln N\right)}^{(3+M)/2}\frac{1}{N^{(1+M)/2}}.$$ (3.25)

We denote by


$$T_{2}\left(\tanh^{2},z_{\iota (i)}^{\left(k\right)}\right)(s):=\tanh^{2}\left(\frac{z_{\iota (i)}^{\left(k\right)}}{\sqrt{\alpha_{\iota (i)}}}\right)+{\left(\tanh^{2}\right)}^{\prime }\left(\frac{z_{\iota (i)}^{\left(k\right)}}{\sqrt{\alpha_{\iota (i)}}}\right)\left(s-\frac{z_{\iota (i)}^{\left(k\right)}}{\sqrt{\alpha_{\iota (i)}}}\right)\;\;+\frac{1}{2}{\left(\tanh^{2}\right)}^{\prime \prime }\left(\frac{z_{\iota (i)}^{\left(k\right)}}{\sqrt{\alpha_{\iota (i)}}}\right){\left(s-\frac{z_{\iota (i)}^{\left(k\right)}}{\sqrt{\alpha_{\iota (i)}}}\right)}^{2}$$

the second order Taylor series of tanh at $z_{\iota (i)}^{\left(k\right)}$. Using that all derivatives of $\tanh^{2}$ are uniformly bounded, Taylor’s Theorem implies that


$$\left|\tanh^{2}(x_{\iota (i)})-T_{2}\left(\tanh^{2},z_{\iota (i)}^{\left(k\right)}\right)\left(x_{\iota (i)}\right)\right|\leq C{\left|x_{\iota (i)}-z_{\iota (i)}^{\left(k\right)}\right|}^{3},$$

and this implies that

$$\;|\;\int_{B\left(z^{\left(k\right)},\rho_{N}\right)}e^{-\frac{N}{2}\langle H_{k}(x-z^{\left(k\right)}),(x-z^{\left(k\right)})\rangle }\tanh^{2}\left(\frac{x_{\iota (i)}}{\sqrt{\alpha_{\iota (i)}}}\right)\;dx\;\;-\int_{B\left(z^{\left(k\right)},\rho_{N}\right)}e^{-\frac{N}{2}\langle H_{k}(x-z^{\left(k\right)}),(x-z^{\left(k\right)})\rangle }T_{2}\left(\tanh^{2},z_{\iota (i)}^{\left(k\right)}\right)\left(\frac{x_{\iota (i)}}{\sqrt{\alpha_{\iota (i)}}}\right)\;dx\;|\;\leq C\int_{B\left(z^{\left(k\right)},\rho_{N}\right)}{\left\|x-z^{\left(k\right)}\right\|}^{3}\;dx\leq Cr_{N}^{3+M}\;\leq C{\left(\ln N\right)}^{(3+M)/2}\frac{1}{N^{(3+M)/2}}.$$ (3.26)

Next, since the function $y\mapsto e^{-N/2\langle H_{k}y,y\rangle }y_{\lambda }$ is odd and its integral vanishes, Proposition 4.2 implies that

$$\;\int_{{\mathbb{R}}^{M}}e^{-\frac{N}{2}\langle H_{k}(x-z^{\left(k\right)}),(x-z^{\left(k\right)})\rangle }T_{2}\left(\tanh^{2},z_{\iota (i)}^{\left(k\right)}\right)\left(x_{\iota (i)}\right)\;dx\;=\sum_{k=1}^{K}\left(\frac{(2\pi {)}^{M/2}}{\det(H_{k}{)}^{1/2}}\frac{1}{N^{M/2}}\right)\left(\tanh^{2}\left(\frac{z_{\iota (i)}^{\left(k\right)}}{\sqrt{\alpha_{\iota (i)}}}\right)+\frac{1}{N}\frac{1}{2}{\left(\tanh^{2}\right)}^{\prime \prime }\left(\frac{z_{\iota (i)}^{\left(k\right)}}{\sqrt{\alpha_{\iota (i)}}}\right){\left(H_{k}^{-1}\right)}_{\iota (i),\iota (i)}\right).$$ (3.27)

It follows from (3.1), (3.20), (3.22), (3.24), (3.26), and (3.27) that

$$\;|Z_{2}(N)-\sum_{k=1}^{K}\left(\frac{(2\pi {)}^{M/2}}{\det(H_{k}{)}^{1/2}}\frac{1}{N^{M/2}}\right)(\tanh^{2}\left(\frac{z_{\iota (i)}^{\left(k\right)}}{\sqrt{\alpha_{\iota (i)}}}\right)\;\;+\frac{1}{N}\frac{1}{2}{\left(\tanh^{2}\right)}^{\prime \prime }\left(\frac{z_{\iota (i)}^{\left(k\right)}}{\sqrt{\alpha_{\iota (i)}}}\right){\left(H_{k}^{-1}\right)}_{\iota (i),\iota (i)})|\;\leq C{\left(\ln N\right)}^{(3+M)/2}\frac{1}{N^{(1+M)/2}}$$ (3.28)

and it follows from (3.2), (3.21), (3.22), (3.25), and Proposition 4.2 that

$$\left|Z_{0}(N)-\sum_{k=1}^{K}\frac{(2\pi {)}^{M/2}}{\det(H_{k}{)}^{1/2}}\frac{1}{N^{M/2}}\right|\leq C{\left(\ln N\right)}^{(3+M)/2}\frac{1}{N^{(1+M)/2}}.$$ (3.29)

Eqs. (3.28) and (3.29) imply that

$$\left|\frac{Z_{2}(N)}{Z_{0}(N)}-\frac{\sum_{k=1}^{K}\frac{1}{\det(H_{k}{)}^{1/2}}(\tanh^{2}\left(\frac{z_{\iota (i)}^{\left(k\right)}}{\sqrt{\alpha_{\iota (i)}}}\right)+\frac{1}{N}\frac{1}{2}{\left(\tanh^{2}\right)}^{\prime \prime }\left(\frac{z_{\iota (i)}^{\left(k\right)}}{\sqrt{\alpha_{\iota (i)}}}\right){\left(H_{k}^{-1}\right)}_{\iota (i),\iota (i)})}{\sum_{k=1}^{K}\frac{1}{\det(H_{k}{)}^{1/2}}}\right|\;\;\leq C{\left(\ln N\right)}^{(3+M)/2}\frac{1}{\sqrt{N}}.$$ (3.30)

Eqs. (3.30) and (3.3) lead to the desired result.

## 4 Auxiliary results

Recall the definitions of *Z*_2_(*N*) and *Z*_0_(*N*) from (3.1) and (3.2) for fixed $i,j\in {\mathbb{N}}_{N}$ with i≠j


$$Z_{2}(N):=Z_{2}(N;i,j):=\int_{{\mathbb{R}}^{M}}\exp\left(-NF(x)\right)\tanh x_{\iota (i)}\tanh x_{\iota (j)}dx$$

and


$$Z_{0}(N):=\int_{{\mathbb{R}}^{M}}\exp\left(-NF(x)\right)dx.$$

The following theorem is a special case of [26, Theorem 32] a result proved for the first time in [30]. We present it here with a short proof for the reader’s convenience.

**Theorem 4.1.** *The following identity holds*


$$𝔼\left(X_{i}X_{j}\right)=\frac{Z_{2}(N)}{Z_{0}(N)},\;i,j\in {\mathbb{N}}_{N},i\neq j.$$

*Proof:* The Gaussian integral $1=\frac{1}{\sqrt{2\pi }}\int e^{-(s-a{)}^{2}/2}\;ds$ implies, expanding the square, that


$$e^{a^{2}/2}=\frac{1}{\sqrt{2\pi }}\int e^{-s^{2}/2+sa}\;ds.$$

Similarly, we obtain for any positive definite matrix $A\in {\mathbb{R}}^{M\times M}$

$$e^{y^{T}Ay/2N}=\frac{\sqrt{\det(A)}}{{\left(2\pi N\right)}^{M/2}}\int e^{-w^{T}Aw/(2N)+w^{T}Ay/N}\;dw\;=\frac{N^{M/2}}{{\left(2\pi \right)}^{M/2}\sqrt{\det(A)}}\int e^{-(N/2)u^{T}A^{-1}u+u^{T}s(x)}\;du,$$ (4.1)

the latter equality being the result of a change of variables *u* = *N*^−1^*Aw*. The first equality is straightforward for diagonal matrices *A* (using the one-dimensional version). Since *A* is self-adjoint, we can diagonalise it: *A* = *U*^*T*^*DU* for an orthogonal matrix *U* and a diagonal matrix *D*. A change of variables z=Uw, gives the desired result (using the diagonal case with *Uy* instead of *y*).

For every


$$x=\left(x_{1},\ldots ,x_{N}\right)\in \{-1,1{\}}^{N},$$

we set


$$s_{l}(x)≔\frac{1}{\sqrt{\alpha_{l}}}\sum_{i\in \iota^{-1}\left(l\right)}x_{i},\;{\tilde{s}}_{l}(x)≔\frac{1}{\sqrt{N}}s_{l}(x),\;s(x)≔{\left(s_{1}(x),\ldots ,s_{M}(x)\right)}^{T},\;\text{and }\tilde{s}(x)≔{\left({\tilde{s}}_{1}(x),\ldots ,{\tilde{s}}_{M}(x)\right)}^{T}.$$

Recall Definition 1.5 and note that


$$e^{-ℍ(x)}=e^{\tilde{s}(x{)}^{T}J\tilde{s}(x)/2}=e^{s(x{)}^{T}Js(x)/\left(2N\right)}.$$

Using (4.1) with *A* = *J* and y=s(x) and the change of variables *u* = *N*^−1^*Jw*, we obtain


$$e^{-ℍ(x)}=\frac{N^{M/2}}{{\left(2\pi \right)}^{M/2}\sqrt{\det(J)}}\int e^{-(N/2)u^{T}J^{-1}u+u^{T}s(x)}\;du.$$

Noting that the partition function *Z* is given by $Z=\sum_{x}e^{-ℍ(x)}$, we get

$$𝔼\left(X_{i}X_{j}\right)=\frac{\int e^{-(N/2)u^{T}J^{-1}u}\sum_{x}x_{i}x_{j}e^{u^{T}s(x)}\;du}{\int e^{-(N/2)u^{T}J^{-1}u}\sum_{x}e^{u^{T}s(x)}\;du}.$$ (4.2)

We finish by proving that the numerator is *Z*_2_(*N*) and the denominator *Z*_0_(*N*). The equation


$$x_{i}x_{j}e^{u^{T}s(x)}=x_{i}e^{u_{\iota (i)}x_{i}/\sqrt{\alpha_{\iota (i)}}}x_{j}e^{u_{\iota (j)}x_{j}/\sqrt{\alpha_{\iota (j)}}}\prod_{k\notin \{i,j\}}e^{u_{\iota (k)}x_{k}/\sqrt{\alpha_{\iota (k)}}}$$

and the associativity and commutativity of addition yield


$$\;\sum_{x}x_{i}x_{j}e^{u^{T}s(x)}\;=\sum_{x_{1}\in \{-1,1\}}\cdots \sum_{x_{N}\in \{-1,1\}}x_{i}x_{j}e^{u^{T}s(x)}\;=\sum_{x_{i}\in \{-1,1\}}x_{i}e^{u_{\iota (i)}x_{i}/\sqrt{\alpha_{\iota (i)}}}\sum_{x_{j}\in \{-1,1\}}x_{j}e^{u_{\iota (j)}x_{j}/\sqrt{\alpha_{\iota (j)}}}\prod_{k\notin \{i,j\}}\sum_{k\in \{-1,1\}}e^{u_{\iota (k)}x_{k}/\sqrt{\alpha_{\iota (k)}}}.$$

Then we note that


$$\sum_{x_{i}\in \{-1,1\}}x_{i}e^{u_{\iota (i)}x_{i}/\sqrt{\alpha_{\iota (i)}}}=e^{u_{\iota (i)}/\sqrt{\alpha_{\iota (i)}}}-e^{-u_{\iota (i)}/\sqrt{\alpha_{\iota (i)}}}\;=2\sinh\left(\frac{u_{\iota (i)}}{\sqrt{\alpha_{\iota (i)}}}\right)=2\tanh\left(\frac{u_{\iota (i)}}{\sqrt{\alpha_{\iota (i)}}}\right)\cosh\left(\frac{u_{\iota (i)}}{\sqrt{\alpha_{\iota (i)}}}\right),$$

since tanht=sinht/cosht for any t∈ℝ. Similarly,


$$\sum_{x_{j}\in \{-1,1\}}x_{j}e^{u_{\iota (j)}x_{j}}=2\tanh\left(\frac{u_{\iota (j)}}{\sqrt{\alpha_{\iota (j)}}}\right)\cosh\left(\frac{u_{\iota (j)}}{\sqrt{\alpha_{\iota (j)}}}\right),$$

and for all k∉{i,j}


$$\sum_{k\in \{-1,1\}}e^{u_{\iota (k)}x_{k}/\sqrt{\alpha_{\iota (k)}}}=e^{u_{\iota (k)}/\sqrt{\alpha_{\iota (k)}}}+e^{-u_{\iota (k)}/\sqrt{\alpha_{\iota (k)}}}=2\cosh\left(\frac{u_{\iota (k)}}{\sqrt{\alpha_{\iota (k)}}}\right).$$

We have


$$\;\int e^{-(N/2)u^{T}J^{-1}u}\sum_{x}x_{i}x_{j}e^{u^{T}s(x)}\;du\;=2^{N}\int e^{-(N/2)u^{T}J^{-1}u}\tanh\left(\frac{u_{\iota (i)}}{\sqrt{\alpha_{\iota (i)}}}\right)\tanh\left(\frac{u_{\iota (j)}}{\sqrt{\alpha_{\iota (j)}}}\right)\prod_{l=1}^{M}\cosh^{N_{l}}\left(\frac{u_{l}}{\sqrt{\alpha_{l}}}\right)\;du\;=2^{N}\int e^{-N\left(\frac{1}{2}u^{T}J^{-1}u-\sum_{l=1}^{M}\alpha_{l}\ln\cosh\left(\frac{u_{l}}{\sqrt{\alpha_{l}}}\right)\right)}\tanh\left(\frac{u_{\iota (i)}}{\sqrt{\alpha_{\iota (i)}}}\right)\tanh\left(\frac{u_{\iota (j)}}{\sqrt{\alpha_{\iota (j)}}}\right)\;du,$$

where we again used that $N_{l}=\alpha_{l}N$ for all $l\in {\mathbb{N}}_{M}$. Similarly, we obtain an expression for the denominator of (4.2):


$$\int e^{-(N/2)u^{T}J^{-1}u}\sum_{x}e^{u^{T}s(x)}\;du=2^{N}\int e^{-N\left(\frac{1}{2}u^{T}J^{-1}u-\sum_{l=1}^{M}\alpha_{l}\ln\cosh\left(\frac{u_{l}}{\sqrt{\alpha_{l}}}\right)\right)}\;du.$$

The result follows by dividing numerator and denominator by 2${\;}^{\text{N}}$. □

The following result pertaining to Gaussian integrals is used repeatedly throughout the proofs. It is an immediate consequence of the definition of a multivariate normal distribution. See, e.g., [31, pp. 176–177] for a reference.

**Proposition 4.2.** *Fix d∈ℕ. We have for all invertible $A\in {\mathbb{R}}^{d\times d}$*


$$\frac{1}{(2\pi {)}^{d/2}}\sqrt{\det(A)}\int e^{-\frac{1}{2}z^{T}Az}z^{T}z\;dz=A^{-1},\;\frac{1}{(2\pi {)}^{d/2}}\sqrt{\det(A)}\int e^{-\frac{1}{2}z^{T}Az}\;dz=1.$$

## Supporting information

S1 Text (PDF)
