# Supplementary material for: Detection of an arbitrary number of communities in a block spin Ising model
Source: PLoS One. 2026 Mar 17;21(3):e0339060. doi: 10.1371/journal.pone.0339060 (PMC12995317; doi:10.1371/journal.pone.0339060)
Supplement: S1 Text — (PDF) [file pone.0339060.s001.pdf]

Supplementary Material to the Article
‘Detection of an Arbitrary Number of Communities in a Block Spin Ising Model’

In this document, we present a concrete example of the reconstruction algorithm applied to a sample of simulated data.

We sample from the distribution of the CWM model

$$\mathbb{P}\left(X_1 = x_1, \ldots, X_N = x_N\right) := Z^{-1} e^{-\mathbb{H}(x_1,\ldots,x_N)}$$

with $M = 5$ groups and each group of size $N_1 = \cdots = N_5 = 3$. The coupling matrix is

$$J = \begin{pmatrix} 0.9 & 0.1 & -0.1 & 0.1 & -0.1 \\ 0.1 & 0.8 & 0.1 & -0.1 & 0.1 \\ -0.1 & 0.1 & 0.7 & 0.1 & 0.3 \\ 0.1 & -0.1 & 0.1 & 0.8 & 0.3 \\ -0.1 & 0.1 & 0.3 & 0.3 & 0.3 \end{pmatrix}.$$

J is positive definite, and since $I - J$ is also positive definite, it is in the high temperature regime. Therefore, the voting behaviour is subject to a high degree of disorder. Despite this disorder, we will show how a sample of 100 observations can suffice to identify the group structure without taking into account the above information concerning the number of groups, their respective sizes, and the coupling matrix of the model.

Now assume that each person in this population answers a questionnaire that explores their preferences concerning what makes for their ideal country to live in, exploring aspects such as geography and climate, nature and environment, culture and society, etc. Each question has two possible answers, which we interpret as -1 and $+1$. The responses of the entire population to each question constitute a voting configuration, i.e. an element of $\Omega_1 = \Xi^{15}$ (recall the notation from Section 2 of the article).

The questionnaire, the Python scripts for the simulation algorithm, and a sample generated and analysed below are available at github.com/gabor-toth-ac/Group-Reconstruction-Example.

To simulate what the answers to the questionnaire might look like for the population described by the model specified above, we generate a sample of $n = 100$ observations of (approximately) i.i.d. realisations of the votes from this probability distribution using Markov Chain Monte Carlo (Metropolis) by the following algorithm:

1. We select a starting voting configuration chosen uniformly at random from the set Ω_1.
2. We run the chain for 300 iterations (chosen to be 20 times the number of voters) before recording the first observation.
3. Once we have $t \in \mathbb{N}_{99}$ observations, we run the chain for another 300 iterations and record observation $t + 1$.

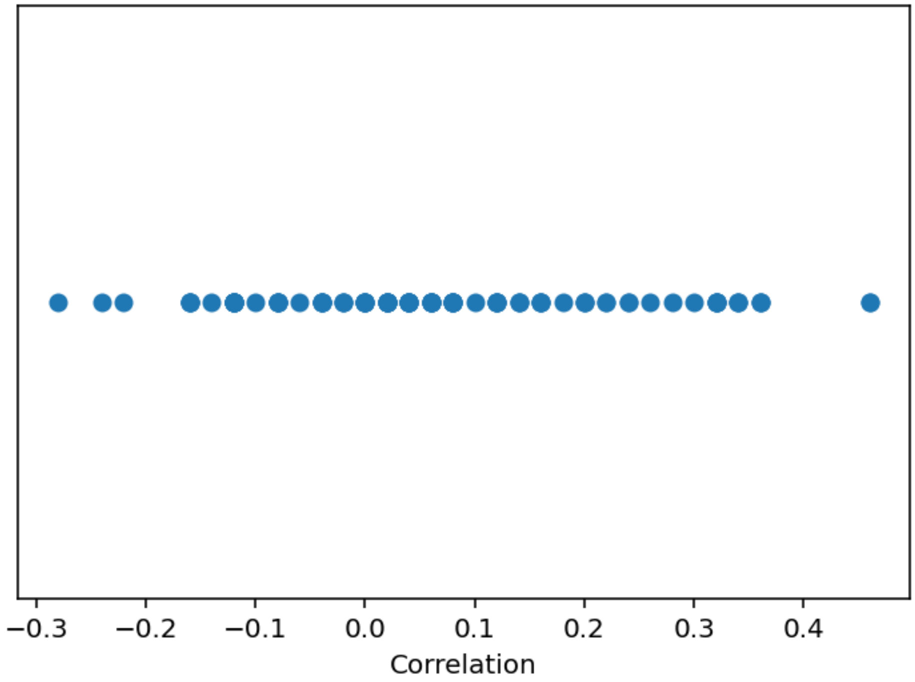

Figure 1: Empirical pair correlations

4. We execute step 3 repeatedly until we have a sample of 100 observations.

The script simulation.py calls functions from the files sampling.py and correlations.py to first generate a sample and then calculate and represent the correlations in a diagram (see Figure 1). The parameters of the model and the sample size are specified in the file simulation.py. A sample of size 100 generated by this procedure is available in the file sample.csv at the above repository for download. We now discuss how to detect the group structure of the underlying model from said sample. We calculate the $\binom{15}{2} = 105$ empirical pair correlations between all possible pairs of voters from the sample, and list them in descending order. Figure 1 visualises the empirical correlations from the sample (each blue dot corresponds to a value taken by at least one correlation), and Table 1 gives the exact values of the correlations and the corresponding identities of the voters. The table on the left side gives the large correlations, and the table on the right gives the smaller correlations.

Notation 1. We will write $\rho_{i,j}$ for the empirical correlation between individuals i and j for all $i, j \in \mathbb{N}_N$.

We will now execute the reconstruction algorithm. By grouping together the correlations in Table 1, we iteratively identify the groups of voters. This is an application of the mathematical work in Section 2.2 of the article to a specific sample.

As a first step, we take a look at the plot of the empirical correlations calculated from the sample (see Figure 1), and we note that there is one high value of empirical correlations to the right. Comparing with the specific values in Table 1, we see there are two correlations $\rho_{10,11}$ and $\rho_{10,12}$ with this value equal to 0.46. Since these correlations are by far higher than all other ones, this is evidence that individuals $10, 11$, and 12 belong to

Indices	Correlations			
(10,11)	0.46			
(10,12)	0.46			
(5,6)	0.36	0.36		
(8,9)	0.36	0.36	0.36	
(11,12)	0.36			
(1,3)	0.34	0.34	0.34	0.34
(13,14)	0.34	0.34	0.34	0.34
(14,15)	0.34	0.34	0.34	0.34
(2,3)	0.32	0.32	0.32	0.32
(4,5)	0.32	0.32		
(4,6)	0.32	0.32		
(7,8)	0.32	0.32	0.32	
(11,15)	0.32			
(1,2)	0.3	0.3	0.3	0.3
(10,14)	0.28			
(12,14)	0.26			
(7,9)	0.24	0.24	0.24	
(12,13)	0.24			
(4,14)	0.22	0.22		
(11,14)	0.22			
(9,12)	0.2	0.2	0.2	
(11,13)	0.2			
(13,15)	0.2	0.2	0.2	0.2
(10,15)	0.18			
(4,9)	0.16	0.16		
(4,15)	0.16	0.16		
(6,13)	0.16	0.16		
(12,15)	0.16			
(1,5)	0.14	0.14		
(9,14)	0.14	0.14	0.14	
(10,13)	0.14			
(2,4)	0.12	0.12		
(2,6)	0.12	0.12		
(3,4)	0.12	0.12		
(5,9)	0.12	0.12		
(7,11)	0.12			
(8,10)	0.1			
(4,8)	0.08	0.08		
(4,13)	0.08	0.08		
(5,7)	0.08	0.08		
(5,8)	0.08	0.08		
(5,13)	0.08	0.08		
(6,9)	0.08	0.08		
(8,12)	0.08			
(1,4)	0.06	0.06		
(1,6)	0.06	0.06		
(1,12)	0.06			
(2,10)	0.06			
(3,10)	0.06			
(7,10)	0.06			
(9,10)	0.06			
(3,11)	0.04			
(4,7)	0.04	0.04		

Indices	Correlations			
(5,15)	0.04	0.04		
(6,7)	0.04	0.04		
(7,15)	0.04	0.04	0.04	
(8,11)	0.04			
(8,15)	0.04	0.04	0.04	
(9,15)	0.04	0.04	0.04	
(1,7)	0.02	0.02	0.02	
(1,9)	0.02	0.02	0.02	
(2,14)	0.02	0.02	0.02	0.02
(4,10)	0.02			
(5,10)	0.02			
(5,14)	0.02	0.02		
(6,10)	0.02			
(6,14)	0.02	0.02		
(1,10)	0			
(2,5)	0	0		
(2,7)	0	0	0	
(2,11)	0			
(3,5)	0	0		
(4,12)	0			
(1,11)	-0.02			
(3,14)	-0.02	-0.02	-0.02	-0.02
(7,14)	-0.02	-0.02	-0.02	
(1,14)	-0.04	-0.04	-0.04	-0.04
(2,15)	-0.04	-0.04	-0.04	-0.04
(5,12)	-0.04			
(6,8)	-0.04	-0.04		
(6,15)	-0.04	-0.04		
(8,14)	-0.06	-0.06	-0.06	
(2,12)	-0.08			
(2,13)	-0.08	-0.08	-0.08	-0.08
(3,12)	-0.08			
(7,12)	-0.08			
(9,11)	-0.08			
(1,13)	-0.1	-0.1	-0.1	-0.1
(2,9)	-0.12	-0.12	-0.12	
(3,6)	-0.12	-0.12		
(3,9)	-0.12	-0.12	-0.12	
(3,13)	-0.12	-0.12	-0.12	-0.12
(3,15)	-0.12	-0.12	-0.12	-0.12
(4,11)	-0.12			
(6,11)	-0.12			
(6,12)	-0.12			
(8,13)	-0.12	-0.12	-0.12	
(1,15)	-0.14	-0.14	-0.14	-0.14
(2,8)	-0.16	-0.16	-0.16	
(3,7)	-0.16	-0.16	-0.16	
(3,8)	-0.16	-0.16	-0.16	
(5,11)	-0.16			
(1,8)	-0.22	-0.22	-0.22	
(9,13)	-0.24	-0.24	-0.24	
(7,13)	-0.28	-0.28	-0.28	

Table 1: List of empirical pair correlations

the same group which we shall call V_1. However, we do not yet know whether there are other members belonging to the same group or not. To make this determination, we consult Table 1 again and see that the next correlation between any $i \in \{10, 11, 12\}$ and any $j \notin \{10, 11, 12\}$ is $\rho_{11,15}$ with a value of 0.32. While this is lower than $\rho_{11,12} = 0.36$, the difference is not sufficient to conclude with enough certainty that 15 does not belong to V_1. To try to ascertain whether 15 belongs to V_1, we take a look at the correlations $\rho_{10,15}$ and $\rho_{12,15}$, and find their values equal 0.18 and 0.16. These values certainly seem much lower than the range of $[0.36, 0.46]$ for the correlations between $\{10, 11, 12\}$, so we discard the possibility that 15 belongs to V_1. The other possible candidate for being a member of V_1 is 14. However, the empirical correlations involving 14 are $\rho_{10,14} = 0.28$, $\rho_{11,14} = 0.22$, and $\rho_{12,14} = 0.26$. These values are also substantially below the range of $[0.36, 0.46]$, and we conclude that 14 likely does not belong to V_1. As a result of this first step, we posit that there is evidence in the sample to suggest that one of the groups is $V_1 = \{10, 11, 12\}$.

We proceed by deleting all empirical correlations from our list which contain at least one member of V_1, and thus obtain column 3 of Table 1. The highest of the remaining correlations is $\rho_{5,6} = 0.36$. So there seems to be a group V_2 with $\{5, 6\} \subset V_2$. Taking a look further down the list, we note $\rho_{4,5} = \rho_{4,6} = 0.32$. This evidence suggests $4 \in V_2$. The next highest correlation between any $i \in \{4, 5, 6\}$ and any $j \notin \{4, 5, 6\}$ is $\rho_{4,14}$ with a value of 0.22. This is quite a bit lower than $\rho_{4,5} = \rho_{4,6} = 0.32$, and also the correlations between 14 and the other two members 5 and 6 are $\rho_{5,14} = \rho_{6,14} = 0.02$. This is strong evidence that 14 does not belong to V_2. Similarly, we can discard that the remaining individuals $1, 2, 3, 7, 8, 9, 13, 15$ belong to V_2, and therefore conclude that there is evidence in the sample that $V_2 = \{4, 5, 6\}$ is one of the groups in the model.

We next delete all empirical correlations from our list which contain at least one member of V_2, and thus obtain column 4 of Table 1. The highest remaining correlation is $\rho_{8,9} = 0.36$. There seems to be a group V_3 with at least two members 8 and 9. We also note that $\rho_{7,8} = 0.32$ and $\rho_{7,9} = 0.24$ suggests that 7 may belong to V_3 as well. Since the next highest correlation involving individual 7 is $\rho_{7,15} = 0.04$ it seems likely that 7 belongs to V_3. Thus we opt for assigning 7 to the same group as 8 and 9, and conclude that $V_3 = \{7, 8, 9\}$ is one of the groups.

We delete all empirical correlations from our list which contain at least one member of V_3, and thus obtain column 5 of Table 1. We inspect the remaining correlations, and we see that $\rho_{1,3} = 0.34$, $\rho_{2,3} = 0.32$, $\rho_{1,2} = 0.3$, providing us with strong evidence that there is a group V_4 with members 1, 2, and 3. Since the next highest correlation with any $i \in \{1, 2, 3\}$ and any $j \notin \{1, 2, 3\}$ is $\rho_{2,14}$ with a value of 0.02, we conclude that there is evidence that V_4 is one of the groups of the model.

By eliminating all correlations involving any members of V_4, we only have the individuals $13, 14$, and 15 left. As $\rho_{13,14} = \rho_{14,15} = 0.34$ are both high in value, we conclude that the last group to be identified is $V_5 = \{13, 14, 15\}$.

For this particular sample, we did not use knowledge about the number of groups and their sizes in order to reconstruct the group structure.
